# A *Lactobacillus* consortium provides insights into the sleep-exercise-microbiome nexus in proof of concept studies of elite athletes and in the general population

**DOI:** 10.1186/s40168-024-01936-4

**Published:** 2025-01-02

**Authors:** Tindaro Bongiovanni, Marina Santiago, Kinga Zielinska, Jonathan Scheiman, Carolina Barsa, Ralf Jäger, Daniela Pinto, Fabio Rinaldi, Giammaria Giuliani, Tullio Senatore, Aleksandar D. Kostic

**Affiliations:** 1 Player Health and Performance, Palermo Football Club, Palermo, Italy; 2 Department of Biomedical and Neuromotor Sciences, University of Bologna, Bologna, Italy; 3 FitBiomics Inc, New York City, NY USA; 4 Increnovo LLC, Whitefish Bay, WI USA; 5 Giuliani SpA, Milan, Italy; 6 Joslin Diabetes Center, Harvard University, Boston, MA USA

**Keywords:** Microbiome, Probiotic, *Lactobacillus*, Oxidative stress, Testosterone, Recovery, Sleep, Fatigue, Energy

## Abstract

**Background:**

The complex relationship among sleep, exercise, and the gut microbiome presents a unique opportunity to improve health and wellness. Here, we conducted the first large-scale investigation into the influence of a novel elite athlete-derived probiotic, consisting of a multi-strain *Lactobacillus* consortium, on sleep quality, exercise recovery, and gut microbiome composition in both elite athletes (*n* = 11) and the general population (*n* = 257).

**Results:**

Our two-phase study design, which included an open-label study followed by a controlled longitudinal study in a professional soccer team, allowed us to identify key interactions between probiotics, the gut microbiome, and the host. In the placebo-controlled study, we observed significant improvements in self-reported sleep quality by 69%, energy levels by 31%, and bowel movements by 37% after probiotic intervention relative to after placebo. These improvements were associated with a significant decrease in D-ROMS (a marker of oxidative stress) and a significantly higher free-testosterone/cortisol ratio. Multi-omics analyses revealed specific changes in microbiome composition and function, potentially providing mechanistic insights into these observed effects.

**Conclusion:**

This study provides novel insights into how a multi-strain *Lactobacillus* probiotic modulates sleep quality, exercise recovery, and gut microbiome composition in both the general population and elite athletes, and introduces potential mechanisms through which this probiotic could be influencing overall health. Our results emphasize the untapped potential of tailored probiotic interventions derived from extremely fit and healthy individuals in improving several aspects of health and performance directly in humans.

Video Abstract

**Supplementary Information:**

The online version contains supplementary material available at 10.1186/s40168-024-01936-4.

## Background

Human health is defined by a complex interdependent network of factors that include intrinsic genetic features as well as acquired habits that include diet; alcohol, tobacco, and drug use; as well as exercise habits. Between intrinsic features and acquired characteristics lies the microbiome. The microbiome is shaped by both our genetics and environment while simultaneously influencing and being influenced by the host’s health as well [1]. The bidirectional nature of health / microbiome interactions makes studies of the microbiome complex [2–5]. Many cross-sectional studies suffer from the chicken and the egg problem: Are the microbiome changes observed in a diseased population the cause of the disease under study or the result of poor health caused by the disease?

Therefore, a new paradigm for understanding the role of the microbiome in human health is needed. Instead of trying to understand the role of the microbiome in health by comparing the microbiomes of healthy and diseases populations, we identify specific populations with a unique set of stressors to the host that create selective pressure on the gut microbiome. That selective pressure will create a unique microbial ecosystem with specific phenotypes that may help the host respond to the stressors or have negative effects on the host. A longitudinal analysis of these populations can uncover novel connections between the microbiome and host phenotypes (both beneficial and detrimental). For example, the gut microbiome adapts to exercise, and the composition of the microbiome changes when one changes from a more sedentary to a more active lifestyle [6–11]. Correlating these changes to host outcomes over time can improve our understanding of the bacterial strains promoting health.

The three *Lactobacillus* strains found in the probiotic under study here (*L. acidophilus* FB0012*, L. plantarum* FB0015*, L. rhamnosus* FB0047*)* were isolated from the guts of elite athletes. Elite athletes have a different microbiota composition than sedentary individuals [12–16], and in fact, elite endurance runners have been shown to have higher levels of *Veillonella* post-workout (a selective pressure) [12]. While *Veillonella* is particularly interesting in this context, as it has the ability to convert lactate, a byproduct of fatigue, into propionate [17], a potential energy source for host cells, other strains such as these lactobacilli may also promote health and performance through alternate mechanisms of action.

To understand the role of the microbiome in host health, it is vital to study them using controlled interventional studies. Even in small populations, by collecting baseline data and understanding what aspects of health change alongside important blood and urine biomarkers, we can begin to tease apart the role of specific strains in human health. We can understand pharmacokinetics—Does the microbe engraft? If so, where? We can understand pharmacodynamics—What effect does the microbe have on the host system? Which biomarkers change? And we can begin to hypothesize the mechanism of action—How does the microbe affect the host and cause the specific effects under study?

Physical and athletic performance is dependent on the microbiome. In vivo rodent models have shown that voluntary exercise as well as run-to-exhaustion can be decreased by depleting the microbiota with antibiotics and restored through fecal microbiota transplant [18]. Here, we describe the first set of studies reporting on the safety and effects of a human athlete-derived probiotic, including an open-label study in healthy human volunteers and a controlled longitudinal study in a professional soccer team, which validates the results of the initial open-label study in terms of improvements in general health, bowel movements, sleep, and energy. We also collected blood and stool from the placebo-controlled study, which has allowed us to perform a detailed multi-omics analysis that has led to some hypotheses and models by which this probiotic exerts its effect in these populations.

## Materials and methods

### Origin of probiotic strains

Samples were collected as described previously [12]. In short, volunteers were provided with a 15-ml falcon tube with a 1-ml pipette tip inserted inside. Volunteers were instructed to dip the pipette tips into soiled toilet tissue, then place them back into the tubes and label the tubes with the date and time of collection. Samples were kept at 4 °C for short-term storage until sample pickup, at which point they were placed into a − 80 °C freezer for long-term storage. Fecal samples were thawed on ice and resuspended in 2–5 ml of PBS. Serial dilutions were made of resuspended samples and then plated onto MRS agar petri dishes reduced to a pH of 5.5 with acetic acid. Plates were incubated under anaerobic conditions at 37 °C for 48 h. Individual bacterial colonies were picked and transferred to 96-deep well plates and grown in MRS liquid culture for 48 h. Bacterial cultures were pelleted for DNA extraction, genome sequencing, and annotation.

### Open-label study

#### Participants

Emails were sent to more than 1000 potential participants who had expressed interest in FitBiomics products and consented to receiving emails, to recruit participants for the study. Of these, 785 filled out the initial survey and were invited into the study. Those that expressed interest were sent a sample of probiotics and surveys to take before, during, and after 2 weeks of supplementation. A total of 257 participants completed all surveys. Of these 257, the average age was 40.9 years (± 14.6 years). Fifty-three percent were female, 46% were male, and 1% chose to not share their gender. The majority of survey respondents were White (80%), followed by Hispanic / Latino (10%), Asian (8%), American Indian / Alaskan Native (4%), Black (3%), Middle-Eastern / North African (2%), and Native Hawaiian / Pacific Islander (1%).On average, the participants exercised 5.6 days per week (± 1.7 days). The majority of respondents (58%) did not follow any particular diet, but many respondents followed common types of restrictive diets including intermittent fasting (13%), gluten-free diet (12%), dairy-free diet (12%), vegetarian diet (5%), vegan diet (5%), paleo diet (4%), pescetarian diet (3%), keto diet (2%), and whole 30 diet (2%). Gastrointestinal disorders were present in this cohort. Twelve percent had a previous irritable bowel syndrome (IBS) diagnosis, 2% had Celiac disease, 2% had inflammatory bowel disease (IBD), and 1% have regular gastroesophageal reflux disease (GERD).

#### Overview

Surveys, consent forms, and marketing materials were provided to participants. All respondents provided informed consent through online forms. The study was performed over 2 weeks and consisted of baseline survey data followed by 2 weeks of supplementation with the probiotic at a low dose (10 billion cfu per capsule) and a high dose (35 billion cfu per capsule). One capsule was ingested orally per day over 2 weeks, total = 14 capsules. Participants filled out online survey questionnaires at the end of the first seven days and at the end of 14 days.

#### Supplementation

Participants were required to take one capsule per day for 14 days. Each capsule contained 10 billion or 35 billion cfu of lyophilized *L. acidophilus* FB0012, *L. plantarum* FB0015, and *L. rhamnosus* FB0047 with dehydrated potato starch. The probiotic strains are encapsulated in acid-resistant capsules and packaged in Alu-Alu blister packs.

#### Surveys

The questionnaires were created using TypeForm (www.typeform.com). Four questionnaires in total were sent to participants. The first was an intake form consisting of 23 demographics, habits, and lifestyle questions. The second was 18 questions focused on understanding the baseline symptom and habit status of that participant that week. The third survey (25 questions) was taken after 1 week of supplementation, and the fourth survey (41 questions) was taken after the second week. Both of these surveys asked questions related to benefits and side effects experienced by the participants. The fourth and final survey also included a variety of questions related to overall experience during the study, willingness to purchase the product, and likelihood of recommending the product.

#### Data processing and statistics

Survey data was exported from Typeform into an Excel spreadsheet. The survey included numerical, categorical, and open-ended questions. Data was processed into a format compatible with analysis using Python 3.6 in a Jupyter Python notebook. Categorical data was converted to numerical data when possible, and open-ended questions were analyzed manually or scanned automatically for key words / phrases and converted into categorical data. Statistically significant differences were calculated using Python’s Numpy and Scipy libraries using the Mafignn–Whitney *U* test and a significance level of < 0.05. Random Forest modeling was performed using the Python Sci-Kit Learn library.

There was no significant difference between arms in terms of whether the participants believed the treatment improved their overall health and wellness (assessed using a chi-square test). However, significant differences in ratings of different benefits post-placebo and post-probiotic were assessed using an independent paired *T*-test, and four of the eight benefits were found to have significant differences (Fig. 2b).

### Controlled longitudinal study

#### Participants

Eleven trained, male elite soccer players (body mass 81.5 ± 2.5 kg, height 1.85 ± 0.2 m, age 26.5 ± 2.0 years) completed this pilot study. No players were taking non-steroidal anti-inflammatory drugs, or had taken antibiotics or probiotic supplements in the preceding 3 months. Soccer players did not have any gastrointestinal disorders, and did not report taking any purposeful ergogenic aids (aside from macronutrients) or supplements for 1 month prior to the study. As this is not a double-blind study, soccer players were blinded to the treatment, but not the researchers. Moreover, this is not a two-arm study but a single-arm longitudinal study with a placebo period followed by a probiotic supplementation period. We used identical capsules for the “probiotics” and “placebo” with no difference in look, smell, or taste. As the capsules were identical, it was impossible for the players to discern which capsules were the probiotics and which ones were the placebo.

#### Overview

The experimental protocol was approved by the Ethical Independent Committee for Clinical, not pharmacological investigation in Genoa (Italy) and followed the ethical standards of the 1964 Declaration of Helsinki (Rif. 2020/12). The protocol was retrospectively registered on ClinicalTrials.gov (registration number: n.NCT06093139). All the volunteers signed an informed consent. All persons gave their informed consent prior to their inclusion in the study and details that might disclose the identity of the subjects under study have been omitted. The study was performed during an overall period of 25 weeks, starting on the 8th November 2021 and completed on the 6th May 2022. In detail, it was a first period of 12 weeks where athletes ingested 4 capsules of placebo per week, followed by 1 week of non-supplementation, and another 12-week period where athletes ingested 4 capsules of probiotics per week.

#### Supplementation

During the first 12-week period (from 8th November 2021 to 6th February 2022), soccer players were required to take one capsule per day for four consecutive days of the placebo capsule (placebo consisted of the same capsules filled with potato starch), then 1 week of non-supplementation, followed by the second period of supplementation (from 14th February 2021 to 6th May 2022) where players were required to take one capsule per day for four consecutive days of the probiotic capsule (a total of 10 billion cfu of a multi-strain probiotic per capsule, consisting of lyophilized *L. acidophilus* FB0012, *L. plantarum* FB0015, and *L. rhamnosus* FB0047) encapsulated in acid-resistant capsules alongside additional potato starch as a filler). The players received probiotics during the week, on the days of their training sessions during the training season. The Club would not change their training schedule, and every player would follow their nutritional periodization plan and a supplementation periodization plan as normal. The players were not aware of the content of the capsules.

#### Short questionnaire

A 12-item short questionnaire was administered after the placebo-supplementation period and after the probiotics-supplementation period. The first nine of these questions consisted of symptoms and benefits experienced by the participants as measured using a numerical rating scale from 1 to 5, where 1 corresponded significantly worse compared to their personal normal, 3 corresponded to no change from personal normal, and 5 corresponded significantly better compared to their personal normal. Two of the final three questions were related to potential adverse effects experienced by the participants, and the final question was on the belief that these probiotics would affect the participant’s overall health and athletic performance.

#### Blood collection and analysis

Venous blood samples were obtained by venipuncture 1 week before the start of this pilot study (baseline values), 1 week after the placebo-supplementation period, and 1 week after the probiotics-supplementation period. Blood samples were centrifuged at 1000 × g for 15 min, and cell-free plasma or serum was aliquoted and stored at − 80 °C until analysis. Plasma 25-hydroxyvitamin D3 was analyzed by chemiluminescent immunoassay on a Roche Elecsys Analyzer 170. The ferritin concentration was measured using the immunoturbidimetric method, on a Roche Cobas Integra 400 biochemical analyzer (Roche Diagnostics, Rotkreuz, Switzerland). Serum cortisol (C) was analyzed with competitive immunoassay using direct chemiluminescence technology (Advia Centaur, Siemens). Free testosterone (FT) was analyzed with ELISA enzyme immunoassay, REF-EIA-29294 (DRG instruments GmbH, Germany); thus the FT:C ratio was obtained. The reactive oxygen metabolites (ROMs) and the biological antioxidant potential (BAP) were measured using the derived ROMs (d-ROMs) and BAP kits, respectively, from Diacron (Grosseto, Italy), according to the manufacturer’s instruction, as previously described (PMID: 26,854,840). TNF-alpha (TNF-α) was measured by ELISA enzyme immunoassay.

#### Fecal metagenomics

Fecal samples were self-collected by each participant using Fe-Col (Alpha Laboratories Ltd, Eastleigh, UK), a disposable paper device to prevent sample contamination, and SMARTeNAT (Copan SpA, Brescia, Italy) for fecal sampling and preservation, as previously done [10]. Samples were stored at − 80 °C within 72 h of collection. One stool sample aliquot was stored raw, while 25 mg was used for DNA extraction. DNA was extracted using the DNeasy Powersoil kit and standard protocols. Shotgun metagenomics analysis of stool samples was performed at Diversigen. Samples underwent Diversigen’s standard quality control (PicoGreen ds DNA quantification) and library preparation. Samples were sequenced on an Illumina NovaSeq 6000 using a 2 × 150-bp flow cell with a mean target depth of 20 million reads.

#### Fecal metabolomics

Metabolomics was performed on ProDigest’s MetaKey global polar metabolomics platform. Raw stool samples were subjected to a solid–liquid-based extraction protocol [19, 20] and subsequently lyophilized. Of lyophilized material, 50.0 ± 1.0 mg was weighed and dissolved in ultrapure water that contained internal standards. After shaking the resulting mixture and performing subsequent centrifugation, the supernatant was collected and filtered through a polyvinylidene fluoride filter (0.22 µm pore size). From the purified extract, a 10-µL aliquot was injected into the ultra high performance liquid chromatography high-resolution mass spectrometry (UHPLC-HRMS) system and performed as described previously [19, 20]. Chromatographic separation was achieved on a Vanquish quaternary pumping system (Thermo Fisher Scientific, USA), equipped with an Acquity HSS T3 C18 column (1.8 µm, 150 × 2.1 mm) (Waters Corporation, UK). A binary solvent system consisting of ultrapure water (solvent A) and acetonitrile (solvent B), both acidified with 0.1% formic acid, was used at a constant flow rate and by applying a gradient profile. Detection was performed on a Q-Exactive™ standalone bench top quadrupole-Orbitrap high-resolution mass spectrometer (Thermo Fisher Scientific, USA), which was preceded by heated electrospray ionization (HESI-II source) in polarity switching mode. The instrument was operated at a resolution of 140,000 full width at half maximum and in full-scan mode (m/z scan range of 53.4–800 Da), meaning that all detected ions were registered whereby no fragmentation was applied.

#### Metagenomics data processing and analysis

DNA sequences were taxonomically classified using the MetaPhlAn2 analysis tool. Functional profiling was carried out using the HUMAnN2 analysis pipeline. The functional tables were filtered to the same subset of samples as the filtered taxa tables. Univariate statistics were used as well. Values below the limit of quantitation (LOQ) were replaced by half of the lowest detected relative abundance above the LOQ. The selection of statistical tests was based on a set of preliminary parametric tests, applied separately. Normality was assessed using Shapiro–Wilk normality tests (cut-off for rejection of normality *p*-value ≤ 0.05), and by reviewing quantile–quantile (Q-Q) plots, homogeneity of variances was checked using Levene’s tests (cut-off for rejection of homogeneity of variances *p*-value ≤ 0.05) and outliers based on *Z*-scores (cut-off of |*Z*-score|> 3.5). As the large majority of metabolites were found to be non-normally distributed, we opted to use Wilcoxon tests to compare treatments, unless otherwise stated.

#### Metabolomics data processing and analysis

Metabolites were identified and quantified using Xcalibur software version 4.0.27.21 (Thermo Fisher Scientific, USA). In general, a metabolite was considered below the LOD/LOQ if the metabolite’s observed peak area was below 1000,000 arbitrary units. Following peak integration, the area ratio was determined for each metabolite by calculating the ratio between the area of the metabolite and that of the most suited internal standard. For the applied multivariate statistical analysis, unsupervised principal component analysis (PCA X) and supervised orthogonal partial least squares discriminant analysis (OPLS-DA) modeling were performed using SIMCA® version 17. We focused on metabolites that were significantly changed compared to baseline and / or compared to placebo but for where there was no significant change between baseline and placebo.

#### Multi-omics analyses

We used Qiime2 [21] to calculate microbiome diversity measures, and chose Shannon entropy as the measure of alpha diversity. Differences in Shannon diversity were determined by the Kruskal–Wallis test (normalized *p*-value ≤ 0.05). Qiime2 longitudinal module, specifically volatility analysis, was used to calculate net average abundance changes of taxa over the course of the study. Correlations between taxa, functions, and metabolites were calculated using Qiime2 SCNIC module (SparCC method) [22]. The significance threshold was set at |R|> 0.1. For every metabolite, a taxonomic-functional cluster was determined, composed of species and functions correlated with the metabolite and with each other. A correlation heatmap was created using Python *seaborn* package. The final correlation network was manually created based on connections of metabolites with similar clusters or depending on their contributions to pathways of interest based on literature sources.

#### Testosterone analysis

To investigate the importance of the 3β-hydroxysteroid dehydrogenase gene and whether its relative abundance was changed during the study, each sample was blasted against the known gene from *Mycolicibacterium neoaurum* (NZ_CP074376.1: 2,036,414–2037514), and the number of hits was compared between baseline, placebo, and probiotic groups. Only hits with a similarity of at least 97% were considered, and the final comparison was made possible by normalizing the hits by the number of sequences in the forward read pair of each sample. A two-tailed *t*-test was performed to determine whether there were significant differences between the groups.

## Results

### Open-label study identifies benefits in sleep and energy

The open-label study was designed to help understand the benefits associated with the supplementation of three athlete-derived probiotic strains (commercially known as Nella and provided by FitBiomics): *L. acidophilus, L. rhamnosus,* and *L. plantarum* encapsulated in acid-resistant delayed-release capsules with potato starch as additional filler. During the study, the participants were supplemented with a high-dose (3.5 × 10^10^ cfu) or low-dose (1 × 10^10^ cfu) probiotic, one oral capsule per day, for 2 weeks. At baseline, 1 week, and 2 weeks, participants were asked to fill out surveys. The baseline survey consisted of baseline habit and lifestyle questions, medical history, demographics, and other questions to help understand the types of participants who were interested in this probiotic. The 1-week and 2-week surveys focused on tracking adverse events (AEs) as well as understanding the benefits that participants were experiencing. The final survey (at 2 weeks) also included final questions on overall experience during probiotic supplementation.

**Fig. 1:**
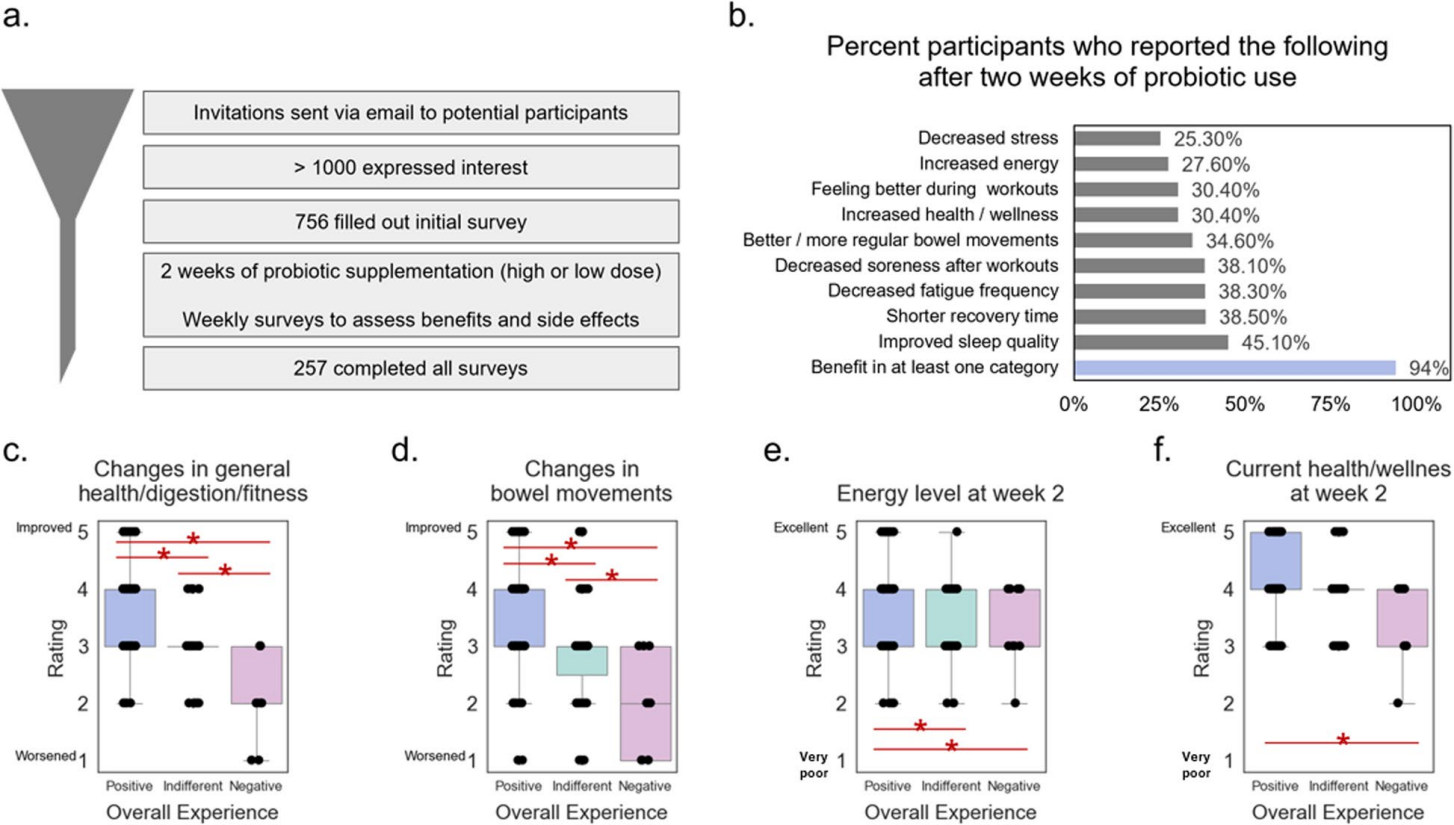
Open-label study was performed to better understand the product benefits. **a** Over 1000 participants were invited to the study. Participants took 1 capsule of probiotic (either a high or low dose) daily for 2 weeks, completing a baseline survey and additional weekly surveys. A total of 257 participants completed the survey. **b** Ninety-four percent of participants reported an improvement in at least one of the benefits described in the survey. The three benefits most often reported included increased sleep quality, shorter recovery time, and decreased frequency of fatigue. **c**–**f** Four features of the random forest model were benefits that had statistically significant differences between high and low overall experience ratings. Statistically significant changes are marked with red stars

More than 1000 participants were invited into the study, and while 756 completed the first survey, 257 completed all surveys (Fig. 1a). Surveys from these 257 were used for analysis. Most AEs were mild, limited, and included symptoms generally associated with probiotic use such as gas, bloating, mild stomach upset, and changes in bowel movements (Additional file 1). These generally subsided by the end of probiotic supplementation. There were no significant differences in AEs between high- and low-dose groups. Of the 257 participants who completed all surveys, 12 (4.7%) left the study due to the side effects that they experienced.

After 2 weeks of probiotic use, 94% of participants reported benefits in at least one of the areas assessed (Fig. 1b). The top five most commonly reported benefits include improved sleep quality (45.1%), shorter recovery time post-exercise (38.5%), decreased frequency of fatigue (38.3%), decreased soreness after workouts (38.1%), and better / more regular bowel movements (34.6%). Participants were also asked to rate their overall experience with the probiotic at the end of the 2 weeks. We used these data to create a model to evaluate which characteristics those that experienced benefits had in common or were driving the reported positive experience. A random forest machine learning model was implemented to identify features that drove the best overall experience (AUC = 0.74). Of the features deemed most important (Additional file 2), four features (general health/digestion/fitness, change in bowel movements, energy level, and current health/wellness) were benefits that had statistically significant differences between high and low overall experience ratings (Fig. 1c–f). These results show that those that had the highest-rated overall experience tended to report better current health, higher energy, better bowel movements, and better general health / digestion / fitness than those that reported a low overall experience.

### Controlled longitudinal study in soccer club used to validate open-label study

**Fig. 2:**
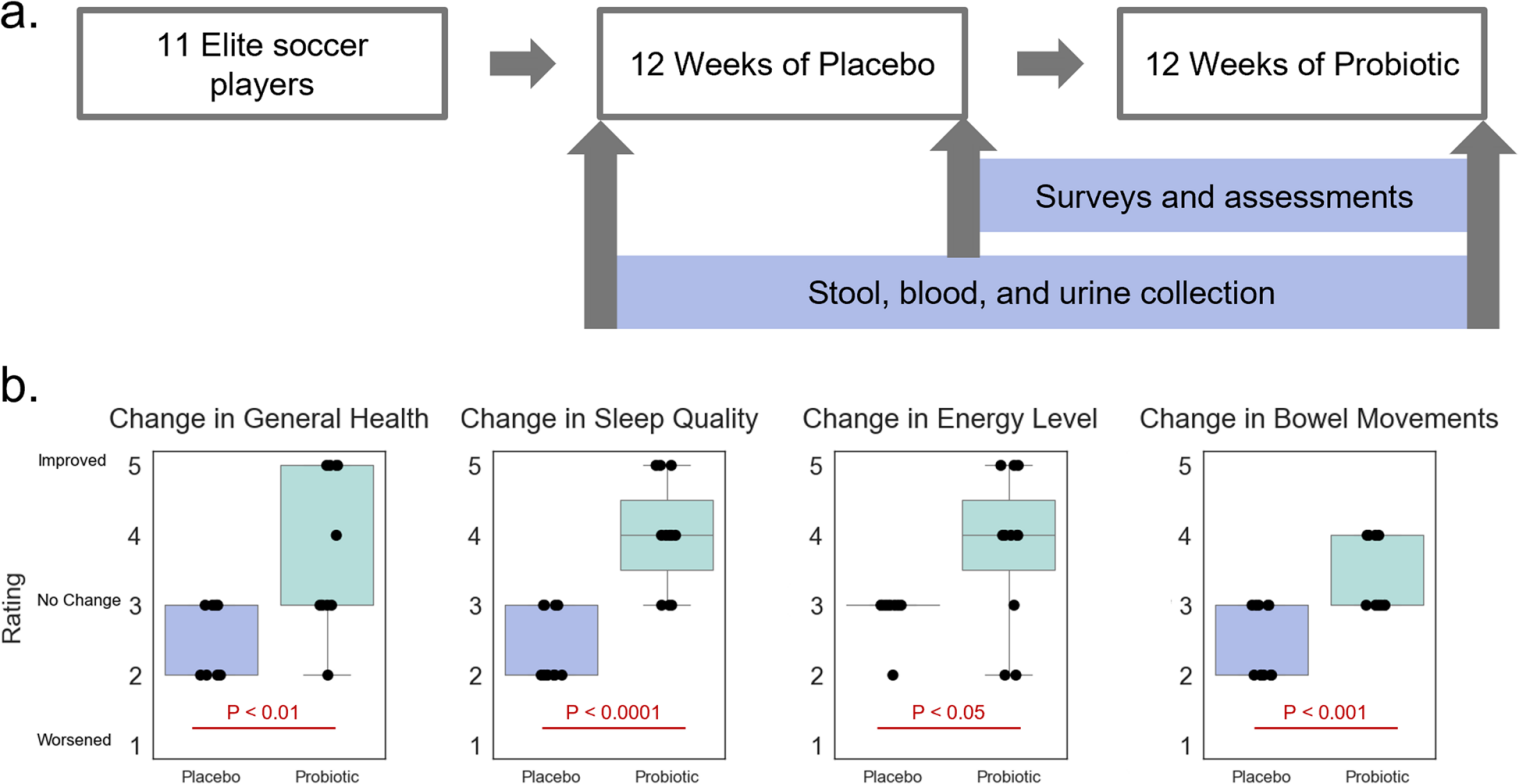
Results of the controlled longitudinal study in a professional sports team. **a** Study participants were recruited from a professional Italian soccer team. Participants were provided with placebo for 12 weeks, followed by a short washout and then probiotic for 12 weeks. Stool, blood, and urine were collected at baseline, after placebo, and after probiotic use. Surveys and assessments were performed after the placebo and after the probiotic. **b** Four benefits were found to be significantly improved after the probiotic compared to the placebo: general health, sleep quality, energy level, and bowel movement quality

To validate the results of the open-label study and further explore the benefits associated with probiotic use, we designed a placebo-controlled longitudinal study and enrolled 15 elite Italian soccer players from a professional SERIE B team. Of the 15 who started the study, 11 completed all aspects over the 24-week study (Fig. 2a). All participants were males (age, 28.7 ± 5.0 years), who performed regular intense exercise as part of the soccer training and competition season. As part of the study, participants took four capsules per week for 24 weeks and were blinded to whether they received placebo or probiotic. During the first 12 weeks, they consumed four placebo capsules per week, and during the second 12 weeks, they consumed four probiotic capsules per week containing 1 × 10^10^ cfu probiotic strains. There was a one-week washout between 12-week periods. Stool, urine, and blood were collected at baseline, post-placebo, and post-probiotic. No significant changes to diet and exercise load occurred during the study period.

A short survey (Additional file 3) was also administered post-placebo, and post-probiotic, which included 8 questions on potential benefits of the probiotic (on a 1–5 rating scale). The survey questions assessed many of the same benefits that the open-label study did. No AEs were reported by participants, and no participant discontinued due to AEs. There was no significant difference between arms in terms of whether the participants believed the treatment improved their overall health and wellness (assessed using a chi-square test). However, significant differences in ratings of different benefits post-placebo and post-probiotic were assessed using an independent paired *T*-test, and four of the eight benefits were found to have significant differences (Fig. 2b). These benefits included a general health rating, improved sleep quality, improved energy level, and improved bowel movements—some of the same benefits that were most commonly reported by the participants in the open-label study. In particular, these results validate the observed improvements in sleep quality and bowel movements, which seem to promote better general health.

### Samples were collected for understanding mechanism of action

**Fig. 3:**
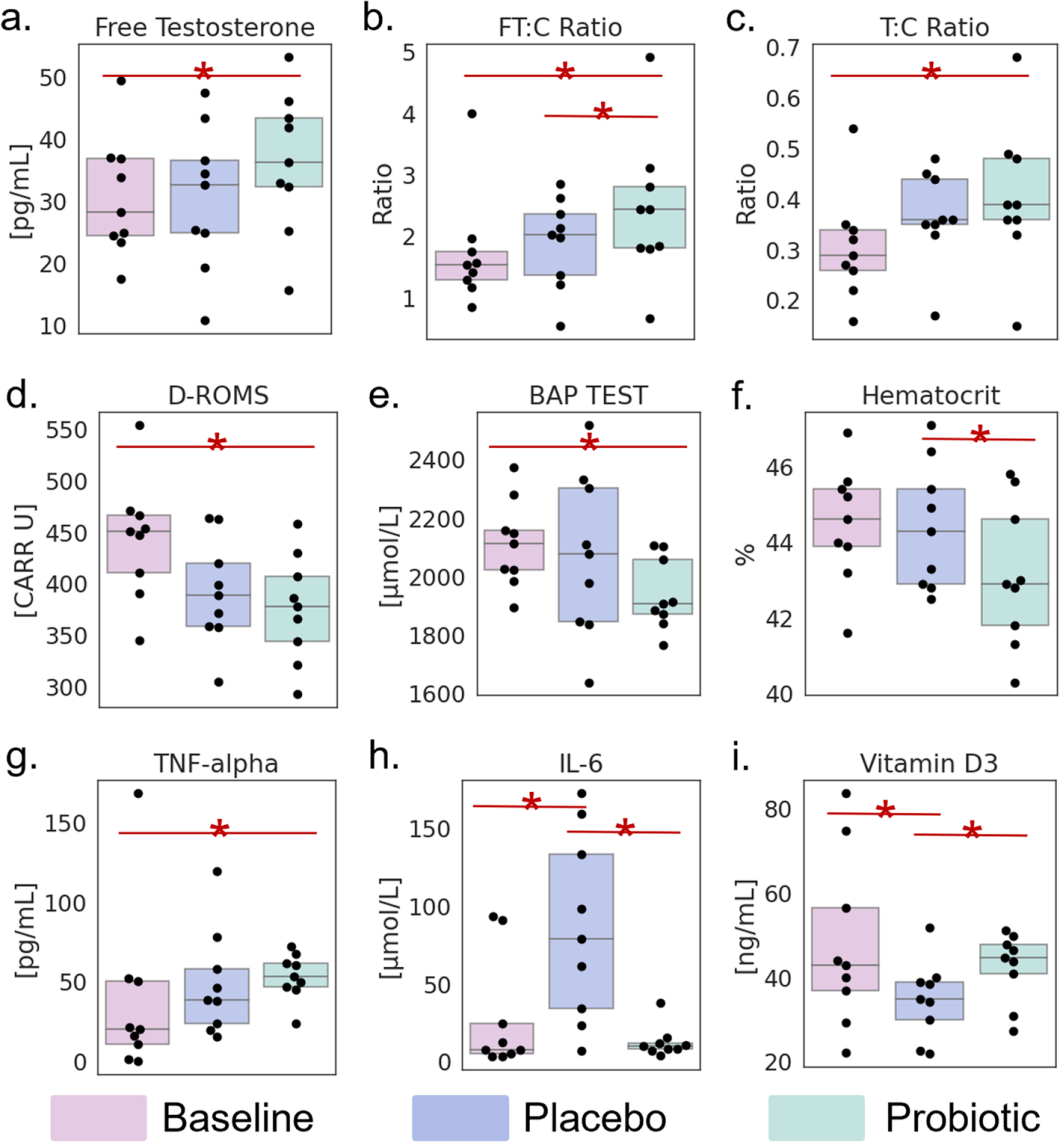
Blood biomarkers that change with probiotic use. Plasma biomarkers were assessed to understand which host biomarkers changed with probiotic use. **a**–**c** Testosterone increases were associated with probiotic use. In particular, free testosterone and the free testosterone to cortisol (FT:C) ratio were increased with probiotic compared to baseline and placebo. Plasma testosterone-to-cortisol ratio (T:C) was only increased with probiotic compared to baseline. **d**, **e** Markers of oxidative stress including derivatives of reactive oxygen metabolites (D-ROMS) and biological antioxidant potential (BAP) appeared to decrease with probiotic use compared to baseline. **f** Hematocrit was decreased with probiotic compared to placebo. **g**, **h** Important cytokines also changed with probiotic use. TNF-alpha was increased with probiotic compared to baseline. IL-6 significantly increased with placebo and decreased with probiotic back to baseline levels. **i** Vitamin D3 also significantly changed at both timepoints, but was decreased with placebo and increased back to baseline levels with probiotic

While probiotics are well-known for their ability to promote better gut health, digestion, and regular bowel movements [23, 24], and some small studies have even found correlations between gut microbiome composition and sleep quality [25–27], and that probiotics may promote sleep quality under specific disease conditions [28, 29], the results of interventional probiotics studies have varied enough that the benefits of probiotics for sleep in the general population and clues to the underlying mechanisms remain an open question [30, 31]. Therefore, we were interested in using the biological samples collected from participants for developing hypotheses for a mechanism by which the probiotic bacteria could potentially improve sleep quality of the host. A panel of 32 plasma biomarkers were chosen for analysis. These biomarkers included general measures of health as well as commonly measured biomarkers associated with exercise and performance. An ANOVA analysis was performed to identify trends (*p* < 0.1) and significant (*p* < 0.05) differences associated with probiotic use as well as markers that simply tend to change over time (Table 1, Fig. 3).

**Table 1:** Plasma biomarkers that changed (significant or trending) with probiotic use

Biomarker	Change observed with placebo	Change observed with probiotic	Main effect *p*-value	*p*-value (T0–T1)	*p*-value (T0–T2)	*p*-value (T1–T2)	Category
Vitamin D3*	Decrease from baseline	Increase from placebo	**0.011**	**0.017**	0.422	** < 0.001**	Other
TNF-alpha*	No change	Increase from baseline	**0.045**	0.321	**0.012**	*0.091*	Oxidative stress / inflammation
IL-6*	Increase from baseline	Decrease from placebo	**0.035**	**0.014**	0.810	**0.024**	Oxidative stress / inflammation
Plasma testosterone	No change	No change	*0.087*	0.574	*0.074*	*0.085*	Recovery from strain
T:C ratio*	No change	Increase from baseline	*0.066*	*0.091*	**0.022**	0.485	Recovery from strain
FT:C ratio*	No change	Increase from baseline and placebo	**0.035**	0.810	**0.014**	**0.024**	Recovery from strain
Free testosterone*	No change	Increase from baseline and placebo	**0.003**	0.560	**0.017**	** < 0.001**	Recovery from strain
Cortisol	Decrease from baseline	No change	*0.076*	**0.023**	0.174	0.303	Recovery from strain
Transferrin saturation (TSAT)	Decrease from baseline	Decrease from baseline	**0.021**	**0.039**	**0.011**	0.875	RBC physiology
MCV	No change	No change	*0.086*	*0.051*	*0.051*	1.000	RBC physiology
Homocysteine	Decrease from baseline	Decrease from baseline	**0.018**	**0.007**	**0.009**	0.897	Other
Iron	Decrease from baseline	No change	**0.036**	**0.028**	*0.058*	0.560	RBC physiology
Hematocrit*	No change	Decrease from placebo	**0.045**	0.847	*0.077*	**0.036**	RBC physiology
Eosinophils	Increase from baseline	Increase from baseline	**0.013**	**0.005**	**0.005**	1.000	RBC physiology
D-ROMS*	No change	Decrease from baseline	**0.005**	*0.052*	**0.001**	0.301	Oxidative stress / inflammation
BAP test*	No change	Decrease from baseline	*0.095*	0.912	**0.015**	*0.054*	Oxidative stress / inflammation

Significant values (*p < 0.05) are bolded, while those with only a trend (*p* < 0.1) are italicized. Starred biomarkers are those with unique changes with probiotic but not placebo. We can roughly classify all these markers into 3 categories: 1) changes to testosterone / cortisol ratio (amount of strain on body / whether getting sufficient recovery); 2) Measures of inflammation and oxidative stress; 3) Changes to red blood cells (iron levels, etc)

*p < 0.05

The majority of the biomarkers with significant changes seemed to be related to (1) oxidative stress / inflammation, (2) recovery from strain, and (3) red blood cell physiology. Markers from these three categories seem to be both changing over time and potentially with probiotic use. Starred biomarkers in Table 1 (and shown in Fig. 3) correspond to those biomarkers with unique changes associated with probiotic use (changes from baseline to probiotic or from placebo to probiotic timepoints but no change or opposing change from baseline to placebo timepoints). Three of these markers are related to testosterone levels: free testosterone, free testosterone-to-cortisol ratio (FT:C), and plasma testosterone-to-cortisol ratio (T:C). These markers, in particular FT:C, are associated with improved recovery from physical strain and could contribute to the increased energy participants reported [32, 33]. Markers of oxidative stress were also changed. Derivatives of reactive oxygen metabolites (D-ROMS) and biological antioxidant potential (BAP) decreased with probiotic use compared to baseline. The hematocrit decreased with probiotic compared to placebo, but the values generally remain within the healthy range for adult males. TNF-alpha and IL-6 are two pro-inflammatory cytokines that also change over time. TNF-alpha increases with probiotics compared to baseline. However, IL-6 displays some remarkable changes. After the placebo period, IL-6 concentration dramatically increases, but after probiotic, it returns to baseline levels. Vitamin D3 displayed a similar but opposite pattern—decreasing during placebo but increasing back to baseline levels with probiotic.

To determine whether any of these biomarker changes were associated with any of the benefits significantly associated with probiotic compared to placebo, we classified participants as responders (reported a benefit) or non-responders (no benefit or worsening of symptoms reported). Due to the small number of participants in the study and the generally high fraction of responders, there were no significant associations between these biomarkers and response.

### Metagenomics and metabolomics analyses were combined to hypothesize mechanisms for survey and biomarker results

**Fig. 4:**
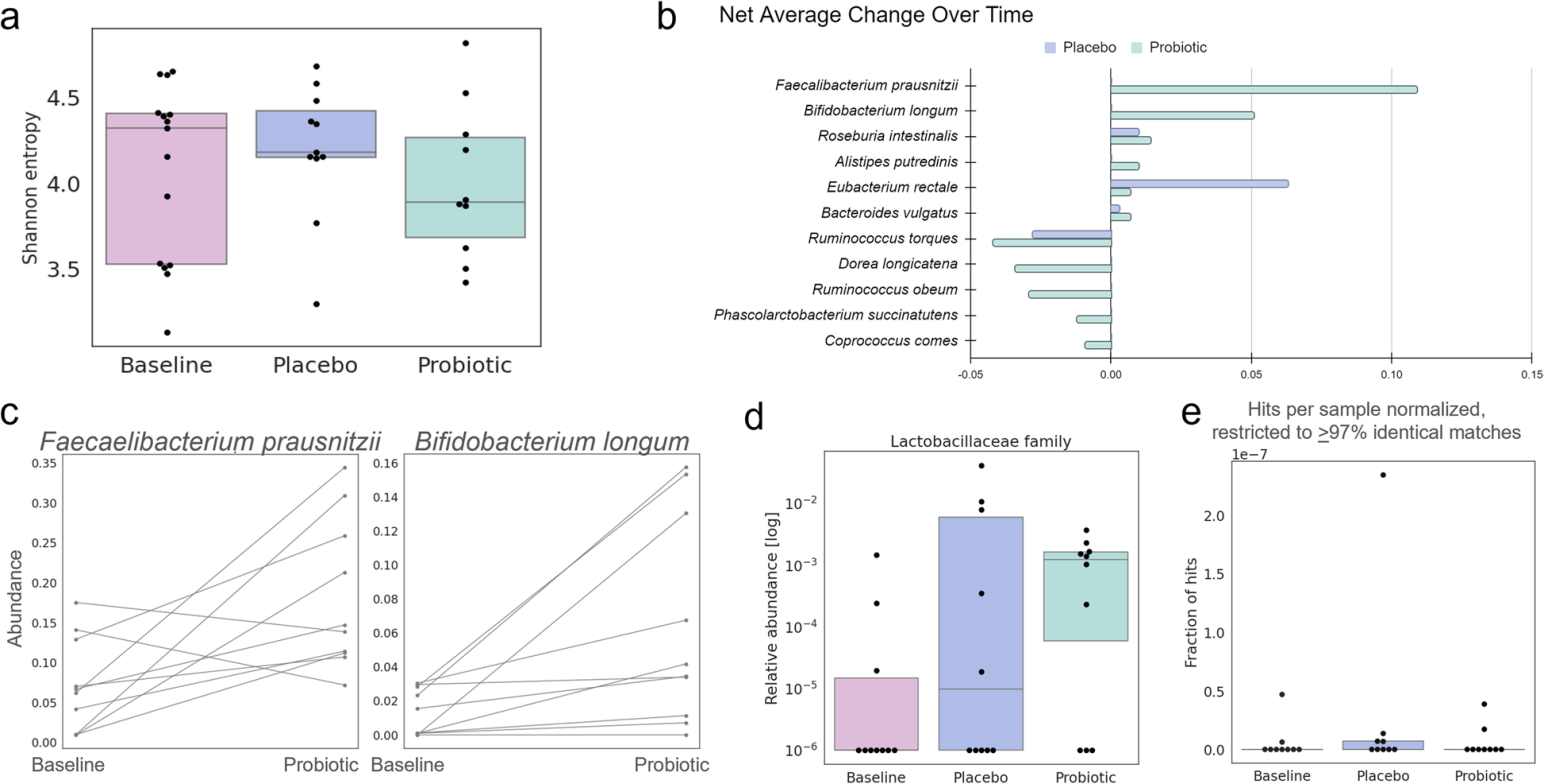
Microbiome community changes over time. **a** There were no changes in alpha diversity over time. **b** A longitudinal analysis of the microbiota community using the longitudinal module of Qiime2 found a list of bacterial species that change over time with placebo and / or with probiotic. **c** These observed changes are generally driven by changes in a subset of participants. Example here shown with *F. prausnitzii*, which increases in most participants over time in contrast with *B. longum*, which increases in only a subset of participants. **d** The abundance of the Lactobacillaceae family increases with probiotic use but not placebo. **e** No significant differences were observed in terms of number of sequences that are similar to 3β-hydroxysteroid dehydrogenase, which has the ability to degrade testosterone

To understand how these biomarkers are being influenced by the gut microbiota, we performed a multi-omics analysis consisting of deep (20 million reads) shotgun metagenomics sequencing and global polar metabolomics profiling of the stool samples provided by the participants. We performed an initial summary analysis of the metagenomics data and used Shannon entropy as a measure of alpha diversity (Fig. 4a). The alpha diversity did not change over time. However, a longitudinal analysis of the data using net average change in abundance calculated as a part of Qiime2’s longitudinal module volatility analysis identified 11 taxa that significantly increased or decreased in relative abundance from baseline (Fig. 4b, Table 2). Of these eleven, seven increased only with probiotic, not with placebo. However, with the exception of *Faecalibacterium prausnitzii*, after plotting these strains individually, the average change over time seems to be driven by a small subset of participants and is not seen generally (Fig. 4c, Additional file 4).

**Table 2:** Taxa that significantly change over time. Bolded taxa change only with probiotic use. Net average change is in terms of relative abundance

Species	Net average change with probiotics	Net average change in placebo
***Faecalibacterium prausnitzii***	**0.109**	**NA**
***Bifidobacterium longum***	**0.051**	**NA**
*Roseburia intestinalis*	0.014	0.010
***Alistipes putredinis***	**0.010**	**NA**
*Eubacterium rectale*	0.007	0.063
*Bacteroides vulgatus*	0.007	0.003
*Ruminococcus torques*	− 0.042	− 0.028
***Dorea longicatena***	− **0.034**	**NA**
***Ruminococcus obeum***	− **0.029**	**NA**
***Phascolarctobacterium succinatutens***	− **0.012**	**NA**
***Coprococcus comes***	− **0.009**	**NA**

To gain a better pharmacokinetic understanding of the probiotic product, we queried the metagenomics data for the species of lactobacilli present in the probiotic (*L. plantarum, L. acidophilus*, and *L. rhamnosus*). While there were no significant changes in the abundance of these species over time, there was a significant increase in the Lactobacillaceae family in general (Fig. 4d). The scientific consensus is that lactobacilli tend to be transient members of the gut community, so it is not surprising that the abundance of these specific species does not change over time with probiotic usage. However, they do promote a healthier gut environment overall, which may explain the slight increase in Lactobacillaceae abundance.

Next, because of the changes in testosterone observed during the study, we investigated whether the microbiome could be modulating testosterone levels. Other studies have found that features of the microbiome such as relative abundance of Firmicutes correlate with testosterone levels in males [34, 35]. Furthermore, 3β-hydroxysteroid dehydrogenase is an enzyme known to be expressed by gut microbes that can degrade testosterone. The abundance of genes encoding this enzyme has been negatively correlated with testosterone levels [36]. Here, we looked for sequences with similarity (97% cut-off) to this gene in the data at baseline, post-placebo, and post-probiotic. We found that there were no significant changes in the fraction of hits at the baseline, placebo, and probiotic timepoints (Fig. 4d). However, in general, there were few hits similar to this sequence. This sequence was chosen due to previous reports suggesting a role of this gene in human testosterone levels, but there may be less similar gene sequences with similar functions that are currently unknown. Furthermore, we may be prevented from observing significant differences due to the small sample size of this study.

The global polar metabolomics analysis yielded 178 metabolites detected above the methodological limit of quantitation. Orthogonal partial least squares discriminant analysis modeling (OPLS-DA) was used to identify metabolites associated with the different timepoints in a supervised manner. Twenty-one metabolites were significantly associated with different timepoints in the study (Additional file 5). We focused our analysis on those 8 metabolites that were found to be important in baseline vs post-probiotic and post-placebo vs post-probiotic but not between baseline and placebo (Table 3).

**Table 3:**
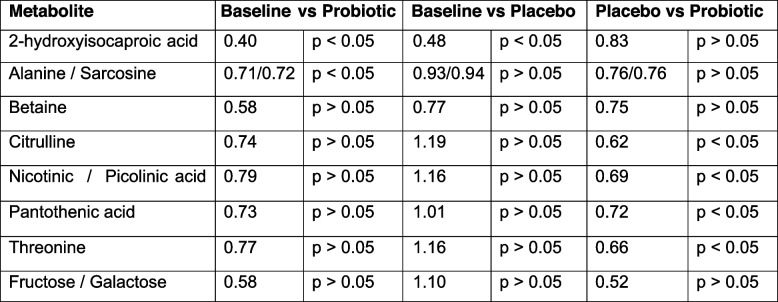
Metabolites that are associated with different timepoints in OPLS-DA modeling

The Variable Importance in Projection (VIP) score is used as the indicator of importance here. A VIP score > 1 indicates a metabolite that is relatively important. All metabolites were found to be decreased in the probiotic condition compared to baseline or to placebo

**Fig. 5:**
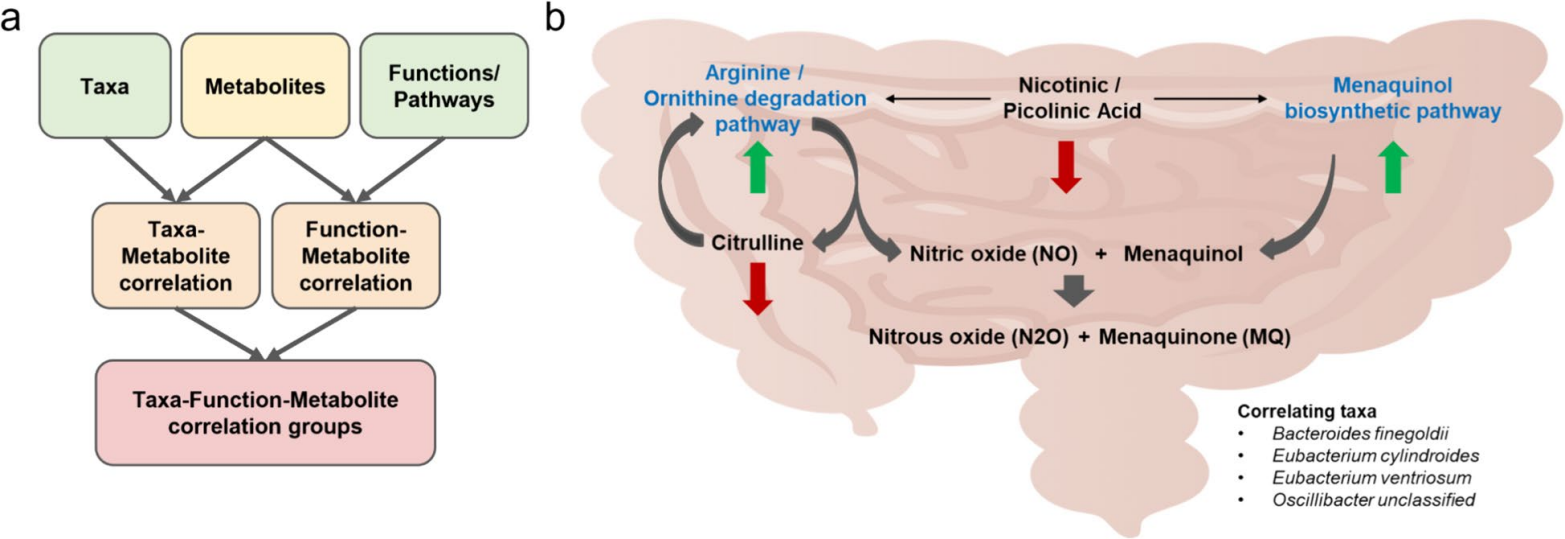
A multi-omics analysis was performed to understand microbiota and metabolite community changes in the athletes. Metabolites that changed with probiotic use but not placebo were identified. **a** The SparCC algorithm was used in a correlational analysis of taxa, pathways, and metabolites that changed over time. In this way, we identified a list of taxa, pathways, and metabolites that correlate with each other. **b**) A biosynthetic pathway approach was used to understand how the different correlating taxa, pathways, and metabolites were related, and a cluster was identified that was associated with menaquinone production and reduced oxidative stress. Nicotinic / picolinic acid is a biosynthetic product of tryptophan. In our data, we found significant correlations between nicotinic / picolinic acid and menaquinol biosynthesis and arginine / ornithine degradation. Arginine / ornithine is metabolized into menaquinol and nitric oxide with citrulline as a byproduct. Menaquinol is converted into menaquinone (vitamin K) and nitrous oxide (N_2_O) Citrulline can be converted back to ornithine to continue this process. The *Bacteroides* genus and some *Eubacterium* species are known producers of menaquinones

In order to understand how these metabolites might be related to changes in the microbiome in terms of both which taxa are represented in the sample as well as which functions and pathways are present, we used a SparCC-based correlational approach (Fig. 5a). First, the eight metabolites that likely changed due to probiotic consumption were correlated with species that changed in the metagenomics data. Similarly, those same eight metabolites were correlated with the functions and pathways present in the metagenomics data. Once we had that list of species as well as a list of functions/pathways correlating with specific metabolomic changes, we assessed which of the taxa and functions correlated with each other.

This resulted in a list of metabolites, taxa, and functions/pathways that were potentially connected to changes due to probiotic use. A clustering-based approach was inconclusive (Additional file 6), so a biosynthetic approach was utilized to understand the relationship between these features. This approach identified a cluster of metabolites and pathways related to oxidative stress (Fig. 5b). Nicotinic acid / picolinic acid (difference could not be determined with the metabolomics methods used) was negatively correlated with arginine and ornithine degradation and menaquinol biosynthesis. Both arginine and ornithine can be broken down to produce NO [37]. Citrulline is another metabolite found to be significantly changed (decreased) with probiotic use. However, citrulline can be recycled back to ornithine. Menaquinol and two molecules of nitric oxide (NO) can be converted into menaquinone, nitrous oxide, and water [38, 39].

**Fig. 6:**
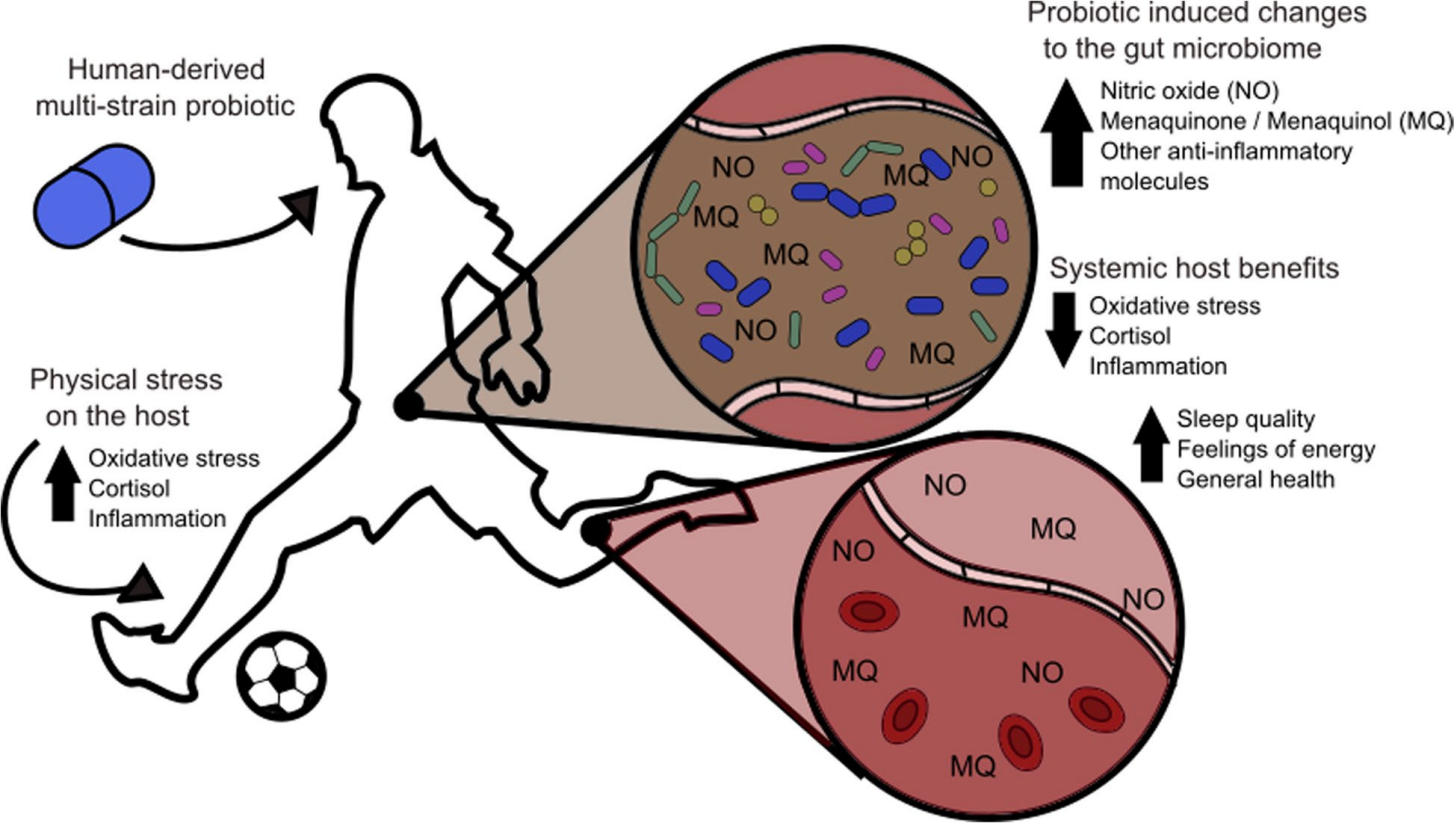
Probiotics provide anti-inflammatory and antioxidant benefits for the host. This probiotic encourages growth or production of menaquinones. An increase in NO and menaquinone (MQ) reduces oxidative stress. Alternatively, outside stressors (exercise, physical strain) induce oxidative stress. The microbiome responds to these changes by changing the composition or regulation of pathways to promote increased production of menaquinones and NO

Menaquinone (vitamin K) is an important nutrient for the human host, with roles in bone, heart, blood, immune, and cognitive health [40–42], and absorption of microbially sourced vitamin K from the small intestine is vital for human health [43]. Both nitric oxide and menaquinone are known antioxidant molecules [44, 45], and microbially modulated nitric oxide has roles in promoting better exercise performance in the mouth microbiome [46]. Nitric oxide may also inhibit cortisol production, which could help explain the testosterone-to-cortisol ratios observed in the biomarker analysis [47]. Four taxa were correlated with these changes: *Bacteroides finegoldii*, *Eubacterium cylindroides*, *Eubacterium ventriosum,* and *Oscillibacter* unclassified. *Bacteroides* species and *Eubacterium lentum* are known biosynthetic producers of menaquinone [48, 49]. Three out of four of the species here are potential menaquinone producers [49]. In the model we hypothesize here (Fig. 6), the probiotic encourages the growth of menaquinone-producing bacteria. An increase in NO and menaquinone modulates oxidative stress, increasing energy and promoting better sleep for the host.

## Discussion

While the gastrointestinal benefits of lactobacilli probiotics are well-known, we observed more novel benefits in these studies in terms of participant-reported improvements in sleep and energy. The gut microbiome’s capacity to adapt to exercise has garnered considerable attention in recent years, with accumulating evidence suggesting that the health benefits of exercise may be mediated by the microbiome [6–9, 12, 18]. The probiotic intervention investigated in this study consists of three lactobacilli strains isolated from elite athletes, whose gut microbiomes have adapted to strenuous exercise. We hypothesize that these exercise-adapted lactobacilli harbor genomic adaptations that contribute to the significant improvements observed in sleep quality, general health, and energy levels after intervention. Moreover, the exercise-adapted microbiome more generally may be responsible for the health improvements conferred by exercise, including sleep quality.

The intricate interplay among sleep, exercise, and the gut microbiome has gained considerable interest in recent years, with a growing body of research illuminating the bidirectional relationship between the gut microbiome and exercise [6–9]. In this study, we investigated the impact of a novel probiotic intervention, developed through the analysis of the gut microbiomes of elite athletes, on sleep quality, exercise recovery, and gut microbiome composition in the general population and in professional athletes.

We observed significant improvements in sleep quality, energy levels, and bowel movement quality after the intervention. Sleep quality and exercise recovery are closely linked, as better sleep contributes to enhanced recovery from physical exertion [50]. The observed improvements in sleep quality and energy suggest that the probiotic intervention could be modulating the host’s physiology and stress response, possibly through the modulation of testosterone, cortisol, and inflammatory markers. Further work is needed to validate these associations and to better understand the underlying mechanisms.

The multi-omics analysis conducted in this study revealed specific changes in blood biomarkers, microbiota, and metabolites associated with the probiotic intervention. Among the observed changes, we found alterations in the levels of specific bacteria such as *F. prausnitzii* and *B. longum*, which have been linked to exercise performance and gut health in previous studies [51, 52]. Additionally, we identified changes in oxidative stress markers, such as the BAP test and D-ROMS, which could be influenced by the gut microbiome [53, 54]. These findings suggest a possible role of the gut microbiome in modulating host physiological responses to exercise and sleep.

Based on these findings and the literature on the intersection of sleep, exercise, and the gut microbiome, we propose a few models of how the exercise-adapted Lactobacilli strains in the probiotic intervention influence sleep quality and other health parameters. One possible model that emerges from our data revolves around the role of oxidative stress in sleep regulation. Sleep quality has been shown to be influenced by the balance between pro- and anti-oxidants in the body [55–57]. As physical exertion increases oxidative stress, the body responds by adjusting antioxidant levels to maintain homeostasis [58]. In addition, the competitive season entails high-intensity peaks, mental stress, and travel. The peaks of BAP test and IL-6, in particular, at placebo point to an external physical stressor, possibly a hard training session or a match within 3–4 days [70].

In our study, we observed changes in biomarkers of oxidative stress including BAP, D-ROMS, cortisol, TNF-α, and IL-6 following the probiotic intervention. However, while BAP and D-ROMS are expected to negatively correlate, we observe decreases in both markers. Because the observed changes are not far outside the normal range for these biomarkers and not particularly associated with an unhealthy state [59, 60], perhaps the decrease in BAP is a result of low D-ROMS. The human host may not require high antioxidant potential when healthy and the levels of reactive oxygen metabolites are low. Our results suggest that the probiotic may enhance sleep quality by modulating the oxidative stress response, ultimately promoting a more balanced redox state conducive to sleep.

Several studies on sleep and inflammation have predominantly measured IL-6 as a marker of systemic inflammation [61–63]. The pattern of values we have observed in IL-6 is consistent with the findings regarding improvement in sleep quality. Sleep disturbances, assessed by symptom reporting with single or multiple items or a questionnaire, correlated with elevated levels of pro-inflammatory cytokines (IL-1, IL-6, TNF-α) [31]. Although it cannot be stated with certainty that the probiotic supplement reduces the level of IL-6, the link found between reduced IL-6 level and improved sleep quality is an important signal for the efficacy of these *Lactobacillus* strains. Especially for *L. plantarum* species, in vivo studies report that it can block the expression of pro-inflammatory cytokines [64]. A recent study in mice also connected changes in IL-6 and TNF-α to oxidative stress in the brain induced by chronic sleep restriction [55]. The authors found that chronic sleep restriction increased TNF-α and reduced IL-6 alongside increased oxidative stress and altered gut-brain hormones. The probiotics used in this study were able to ameliorate the oxidative damage to the brain induced by sleep restriction, reduced neuroinflammation, and positively regulated gut-brain hormones. It remains to be seen whether these effects also occur in humans, but the results of this study suggest that a similar mechanism may be relevant.

Another potential model involves the impact of the probiotic intervention on the production of neurotransmitters and other sleep-regulating molecules. Certain gut microbes are known to produce neuroactive compounds, such as gamma-aminobutyric acid (GABA) and seratonin, which can influence sleep quality [65, 66]. The observed changes in the microbiome community, particularly the increase in taxa, such as *F. prausnitzii* and *B. longum*, may lead to alterations in the production of these neuroactive compounds. This, in turn, could modulate sleep–wake cycles and improve sleep quality.

Furthermore, our study revealed changes in testosterone and cortisol levels following the probiotic intervention. The testosterone-to-cortisol (T:C) ratio is an important indicator of an athlete’s recovery status and is influenced by the balance between anabolic and catabolic processes [32, 33]. An increase in the T:C ratio may signify enhanced recovery and reduced stress, ultimately leading to better sleep quality. The probiotic intervention may impact the host’s hormonal balance, possibly through the modulation of microbial metabolites and their effects on the endocrine system, which in turn influences sleep quality. On the other hand, cortisol and oxidative stress have been shown to be negatively correlated [47]. Perhaps the increased FT:C and T:C ratios are another marker of improved oxidate status of the participants. In fact, testosterone, cortisol, oxidative stress, and even vitamin D (another marker found to improve with probiotic compared to placebo in this study) have previously been shown to be connected in other studies of soccer players [67, 68].

Lastly, the probiotic’s impact on sleep quality could be related to its effects on the immune system. The gut microbiome is known to play a crucial role in the regulation of both innate and adaptive immunity [69]. The observed changes in inflammatory markers, such as TNF-α and IL-6, suggest that the probiotic intervention may modulate the immune response in a way that promotes a more restful sleep. In previous studies, while there tends to be a negative correlation between pro-inflammatory cytokines and improved sleep (as we observe with IL-6), neither sleep disturbance nor sleep duration are generally associated with changes to TNF-α, which we observe to be increased with probiotic use. In the recent study connecting chronic sleep restriction to oxidative stress and neuroinflammation in mice, TNF-α levels are increased with sleep restriction, while IL-6 is decreased [55], which is the same pattern we observe in participants’ post-probiotic use, which is associated with improved sleep quality. More work is needed here to tease apart the relationship between sleep and changes in these inflammatory markers.

There are several limitations to these studies. In the longitudinal placebo-controlled trial, the greatest limitation is the sample size: Only 11 participants completed all aspects of the study. This has limited our ability to identify significant associations between changes to the microbiome, blood-based biomarkers, and participant-reported outcomes. Future research should include larger randomized controlled studies to investigate these effects with greater statistical power. Another limitation was the study design. This study was initially designed as a randomized controlled crossover study, but the logistics of working with a professional soccer club during competition season necessitated a simpler study design. The longitudinal aspect of the study introduces potential confounding variables such as changing seasons, which could explain the changes observed in vitamin D3. Other unknown confounders may explain some of the observed biomarker and microbiome changes as well. While participants were instructed to maintain the same diet, exercise, and lifestyle habits throughout the study, it is impossible to retrospectively determine whether this was the case. Future randomized controlled parallel arm or crossover studies will limit these confounding variables and validate the effects observed here. A major limitation of the open-label study of 257 individuals was that it lacked a control arm, making it susceptible to bias from the placebo effect. However, the results from the open-label study were critical in the design of the placebo-controlled trial, without which we would have not identified the association with sleep quality. Another limitation of the manuscript is that there is no non-survey data related to the effect on the general population; therefore, we cannot confirm that the same effects are present in elite athletes and the general population.

We believe that this work can serve as a model for future clinical investigations of probiotics and other supplements, beginning with a large but low-cost survey-based study exploring a large range of effects that may be conferred by the probiotic. Then any significant effects identified in the large study, serving as hypotheses, can be independently tested and validated in smaller but rigorous placebo-controlled trials.

## Conclusion

In conclusion, we have used a two-phase study approach to identify the potential benefits of a multi-strain *Lactobacillus* probiotic and validated those results using a controlled longitudinal study in an athlete population. These studies found not only improvements in digestion but also significant increases in energy and improved sleep quality. To delve into why this might be, we used multi-omics techniques alongside a panel of blood biomarkers and participant-reported survey results to identify a potential mechanism by which this probiotic could be modulating sleep and have proposed a model by which the probiotic modulates the gut to promote decreased oxidative stress for the host.

## Supplementary Information

Additional file 1: AEs reported during the open-label study.

Additional file 2: Top 10 most important features of the model.

Additional file 3: Survey used in placebo-controlled study.

Additional file 4: Species with significant changes over time.

Additional file 5: Metabolites that discriminate between different timepoints.

Additional file 6: Clustering heatmap of all taxa, pathways, and metabolites.

Additional file 7: Bray–Curtis analysis.

## Data Availability

The metagenomics datasets generated and analyzed during the current study are available in the SRA repository, at accession number PRJNA975983.

## Abbreviations

BAP: Biological antioxidant potential
D-ROMS: Derivatives of reactive oxygen metabolites
FT:C: Free testosterone-to-cortisol ratio
T:C: Plasma testosterone-to-cortisol ratio
VIP: Variable importance in projection
OPLS-DA: Orthogonal partial least squares discriminant analysis
MQ: Menaquinone

## References

[1] Hou K, Wu Z-X, Chen X-Y, Wang J-Q, Zhang D, Xiao C, et al. Microbiota in health and diseases. Signal Transduct Target Ther. 2022;7:1–28. 35461318 10.1038/s41392-022-00974-4 PMC9034083

[2] Carabotti M, Scirocco A, Maselli MA, Severi C. The gut-brain axis: interactions between enteric microbiota, central and enteric nervous systems. Ann Gastroenterol Q Publ Hell Soc Gastroenterol. 2015;28:203–9. PMC4367209 25830558

[3] Dutta D, Lim SH. Bidirectional interaction between intestinal microbiome and cancer: opportunities for therapeutic interventions. Biomark Res. 2020;8:31. 32817793 10.1186/s40364-020-00211-6 PMC7424681

[4] Wang X, Chen Z, Geng B, Cai J. The bidirectional signal communication of microbiota-gut-brain axis in hypertension. Int J Hypertens. 2021;2021:8174789. 34970454 10.1155/2021/8174789 PMC8714396

[5] Zheng D, Liwinski T, Elinav E. Interaction between microbiota and immunity in health and disease. Cell Res. 2020;30:492–506. 32433595 10.1038/s41422-020-0332-7 PMC7264227

[6] Zhang L, Liu Y, Wang X, Zhang X. Physical exercise and diet: regulation of gut microbiota to prevent and treat metabolic disorders to maintain health. Nutrients. 2023;15:1539. 36986268 10.3390/nu15061539 PMC10054346

[7] Boytar AN, Skinner TL, Wallen RE, Jenkins DG, Dekker NM. The effect of exercise prescription on the human gut microbiota and comparison between clinical and apparently healthy populations: a systematic review. Nutrients. 2023;15:1534. 36986264 10.3390/nu15061534 PMC10054511

[8] O’Brien MT, O’Sullivan O, Claesson MJ, Cotter PD. The athlete gut microbiome and its relevance to health and performance: a review. Sports Med Auckl NZ. 2022;52(Suppl 1):119–28. 10.1007/s40279-022-01785-x PMC9734205 36396898

[9] Sales KM, Reimer RA. Unlocking a novel determinant of athletic performance: the role of the gut microbiota, short-chain fatty acids, and “biotics” in exercise. J Sport Health Sci. 2023;12:36–44. 36089243 10.1016/j.jshs.2022.09.002 PMC9923434

[10] Barone M, Bongiovanni T, Quercia S, Di Gesu R, Pasta G, et al. Fecal microbiota monitoring in elite soccer players along the 2019–2020 competitive season. Int J Sports Med. 2022;43:1137–47. 35595508 10.1055/a-1858-1810

[11] Bongiovanni T, Yin MOL, Heaney LM. The athlete and gut microbiome: short-chain fatty acids as potential ergogenic aids for exercise and training. Int J Sports Med. 2021;42:1143–58. 34256388 10.1055/a-1524-2095

[12] Scheiman J, Luber JM, Chavkin TA, MacDonald T, Tung A, Pham L-D, et al. Meta’omic analysis of elite athletes identifies a performance-enhancing microbe that functions via lactate metabolism. Nat Med. 2019;25:1104–9. 31235964 10.1038/s41591-019-0485-4 PMC7368972

[13] Fontana F, Longhi G, Tarracchini C, Mancabelli L, Lugli GA, Alessandri G, et al. The human gut microbiome of athletes: metagenomic and metabolic insights. Microbiome. 2023;11:27. 36782241 10.1186/s40168-023-01470-9 PMC9926762

[14] Kulecka M, Fraczek B, Balabas A, Czarnowski P, Zeber-Lubecka N, Zapala B, et al. Characteristics of the gut microbiome in esports players compared with those in physical education students and professional athletes. Front Nutr. 2022;9:1092846. 36726816 10.3389/fnut.2022.1092846 PMC9884692

[15] Dziewiecka H, Buttar HS, Kasperska A, Ostapiuk-Karolczuk J, Domagalska M, Cichoń J, et al. Physical activity induced alterations of gut microbiota in humans: a systematic review. BMC Sports Sci Med Rehabil. 2022;14:122. 35799284 10.1186/s13102-022-00513-2 PMC9264679

[16] Xu Y, Zhong F, Zheng X, Lai H-Y, Wu C, Huang C. Disparity of gut microbiota composition among elite athletes and young adults with different physical activity independent of dietary status: a matching study. Front Nutr. 2022;9: 843076. 35369075 10.3389/fnut.2022.843076 PMC8975590

[17] Ng SK, Hamilton IR. Carbon dioxide fixation by Veillonella parvula M 4 and its relation to propionic acid formation. Can J Microbiol. 1973;19:715–23. 4712506 10.1139/m73-116

[18] Dohnalová L, Lundgren P, Carty JRE, Goldstein N, Wenski SL, Nanudorn P, et al. A microbiome-dependent gut–brain pathway regulates motivation for exercise. Nature. 2022;612:739–47. 36517598 10.1038/s41586-022-05525-z PMC11162758

[19] Vanden Bussche J, Marzorati M, Laukens D, Vanhaecke L. Validated high resolution mass spectrometry-based approach for metabolomic fingerprinting of the human gut phenotype. Anal Chem. 2015;87:10927–34. 26451617 10.1021/acs.analchem.5b02688

[20] De Paepe E, Van Meulebroek L, Rombouts C, Huysman S, Verplanken K, Lapauw B, et al. A validated multi-matrix platform for metabolomic fingerprinting of human urine, feces and plasma using ultra-high performance liquid-chromatography coupled to hybrid orbitrap high-resolution mass spectrometry. Anal Chim Acta. 2018;1033:108–18. 30172316 10.1016/j.aca.2018.06.065

[21] Bolyen E, Rideout JR, Dillon MR, Bokulich NA, Abnet CC, Al-Ghalith GA, et al. Reproducible, interactive, scalable and extensible microbiome data science using QIIME 2. Nat Biotechnol. 2019;37:852–7. 31341288 10.1038/s41587-019-0209-9 PMC7015180

[22] SCNIC: Sparse Correlation Network Investigation for Compositional Data | bioRxiv. https://www.biorxiv.org/content/10.1101/2020.11.13.380733v1.full. Accessed 11 May 2023. 10.1111/1755-0998.13704 PMC9744196 36001047

[23] Butel M-J. Probiotics, gut microbiota and health. Médecine Mal Infect. 2014;44:1–8. 10.1016/j.medmal.2013.10.002 24290962

[24] Bodke H, Jogdand S. Role of probiotics in human health. Cureus. 2022;14:e31313. 36514580 10.7759/cureus.31313 PMC9733784

[25] Zhang J, Zhang X, Zhang K, Lu X, Yuan G, Yang H, et al. The component and functional pathways of gut microbiota are altered in populations with poor sleep quality - a preliminary report. Pol J Microbiol. 2022;71:241–50. 35716170 10.33073/pjm-2022-021 PMC9252145

[26] Haimov I, Magzal F, Tamir S, Lalzar M, Asraf K, Milman U, et al. Variation in gut microbiota composition is associated with sleep quality and cognitive performance in older adults with insomnia. Nat Sci Sleep. 2022;14:1753–67. 36225322 10.2147/NSS.S377114 PMC9550024

[27] Qiu L, Gong F, Wu J, You D, Zhao Y, Xu L, et al. Exercise interventions improved sleep quality through regulating intestinal microbiota composition. Int J Environ Res Public Health. 2022;19:12385. 36231686 10.3390/ijerph191912385 PMC9564517

[28] Estaki M, Langsetmo L, Shardell M, Mischel A, Jiang L, Zhong Y, et al. Association of subjective and objective measures of sleep with gut microbiota composition and diversity in older men: the Osteoporotic Fractures in Men (MrOS) study. J Gerontol A Biol Sci Med Sci. 2023;78(10):glad011. 10.1093/gerona/glad011 PMC10562887 36655399

[29] Dhiman RK, Rana B, Agrawal S, Garg A, Chopra M, Thumburu KK, et al. Probiotic VSL#3 reduces liver disease severity and hospitalization in patients with cirrhosis: a randomized, controlled trial. Gastroenterology. 2014;147:1327–37.e3. 25450083 10.1053/j.gastro.2014.08.031

[30] Gil-Hernández E, Ruiz-González C, Rodriguez-Arrastia M, Ropero-Padilla C, Rueda-Ruzafa L, Sánchez-Labraca N, et al. Effect of gut microbiota modulation on sleep: a systematic review and meta-analysis of clinical trials. Nutr Rev. 2023;81(12):1556–70. 10.1093/nutrit/nuad027 37023468

[31] Irwin MR, Olmstead R, Carroll JE. Sleep disturbance, sleep duration, and inflammation: a systematic review and meta-analysis of cohort studies and experimental sleep deprivation. Biol Psychiatry. 2016;80:40–52. 26140821 10.1016/j.biopsych.2015.05.014 PMC4666828

[32] Urhausen A, Gabriel H, Kindermann W. Blood hormones as markers of training stress and overtraining. Sports Med Auckl NZ. 1995;20:251–76. 10.2165/00007256-199520040-00004 8584849

[33] Jürimäe J, Jürimäe T, Purge P. Plasma testosterone and cortisol responses to prolonged sculling in male competitive rowers. J Sports Sci. 2001;19:893–8. 11695511 10.1080/026404101753113840

[34] Matsushita M, Fujita K, Motooka D, Hatano K, Hata J, Nishimoto M, et al. Firmicutes in gut microbiota correlate with blood testosterone levels in elderly men. World J Mens Health. 2022;40:517–25. 35274505 10.5534/wjmh.210190 PMC9253793

[35] Colldén H, Landin A, Wallenius V, Elebring E, Fändriks L, Nilsson ME, et al. The gut microbiota is a major regulator of androgen metabolism in intestinal contents. Am J Physiol-Endocrinol Metab. 2019;317:E1182–92. 31689143 10.1152/ajpendo.00338.2019 PMC6962501

[36] Li D, Liu R, Wang M, Peng R, Fu S, Fu A, et al. 3β-Hydroxysteroid dehydrogenase expressed by gut microbes degrades testosterone and is linked to depression in males. Cell Host Microbe. 2022;30:329–339.e5. 35108497 10.1016/j.chom.2022.01.001

[37] Arena ME, Saguir FM, Manca de Nadra MC. Arginine, citrulline and ornithine metabolism by lactic acid bacteria from wine. Int J Food Microbiol. 1999;52:155–61. 10733246 10.1016/s0168-1605(99)00133-6

[38] Cramm R, Pohlmann A, Friedrich B. Purification and characterization of the single-component nitric oxide reductase from Ralstonia eutropha H16. FEBS Lett. 1999;460:6–10. 10571051 10.1016/s0014-5793(99)01315-0

[39] Suharti, Strampraad MJ, Schröder I, de Vries S. A novel copper A containing menaquinol NO reductase from Bacillus azotoformans. Biochemistry. 2001;40:2632–9. 10.1021/bi0020067 11327887

[40] Kidd PM. Vitamins D and K as pleiotropic nutrients: clinical importance to the skeletal and cardiovascular systems and preliminary evidence for synergy. Altern Med Rev J Clin Ther. 2010;15:199–222. 21155624

[41] Shiraki M, Tsugawa N, Okano T. Recent advances in vitamin K-dependent Gla-containing proteins and vitamin K nutrition. Osteoporos Sarcopenia. 2015;1:22–38.

[42] Presse N, Belleville S, Gaudreau P, Greenwood CE, Kergoat M-J, Morais JA, et al. Vitamin K status and cognitive function in healthy older adults. Neurobiol Aging. 2013;34:2777–83. 23850343 10.1016/j.neurobiolaging.2013.05.031

[43] Conly JM, Stein K, Worobetz L, Rutledge-Harding S. The contribution of vitamin K2 (menaquinones) produced by the intestinal microflora to human nutritional requirements for vitamin K. Am J Gastroenterol. 1994;89:915–23. 8198105

[44] Vervoort LMT, Ronden JE, Thijssen HHW. The potent antioxidant activity of the vitamin K cycle in microsomal lipid peroxidation. Biochem Pharmacol. 1997;54:871–6. 9354587 10.1016/s0006-2952(97)00254-2

[45] Hummel SG, Fischer AJ, Martin SM, Schafer FQ, Buettner GR. Nitric oxide as a cellular antioxidant: a little goes a long way. Free Radic Biol Med. 2006;40:501–6. 16443165 10.1016/j.freeradbiomed.2005.08.047 PMC2258411

[46] Bryan NS, Burleigh MC, Easton C. The oral microbiome, nitric oxide and exercise performance. Nitric Oxide. 2022;125–126:23–30. 35636654 10.1016/j.niox.2022.05.004

[47] Monau TR, Vargas VE, Zhang L, Myers DA, Ducsay CA. Nitric oxide inhibits ACTH-induced cortisol production in near-term, long-term hypoxic ovine fetal adrenocortical cells. Reprod Sci Thousand Oaks Calif. 2010;17:955–62. 10.1177/1933719110376092 PMC2943550 20713972

[48] Ramotar K, Conly JM, Chubb H, Louie TJ. Production of menaquinones by intestinal anaerobes. J Infect Dis. 1984;150:213–8. 6470528 10.1093/infdis/150.2.213

[49] Collins MD, Fernandez F, Howarth OW. Isolation and characterization of a novel vitamin-K from Eubacterium lentum. Biochem Biophys Res Commun. 1985;133:322–8. 4074371 10.1016/0006-291x(85)91878-9

[50] Fullagar HHK, Skorski S, Duffield R, Hammes D, Coutts AJ, Meyer T. Sleep and athletic performance: the effects of sleep loss on exercise performance, and physiological and cognitive responses to exercise. Sports Med Auckl NZ. 2015;45:161–86. 10.1007/s40279-014-0260-0 25315456

[51] Barton W, Penney NC, Cronin O, Garcia-Perez I, Molloy MG, Holmes E, et al. The microbiome of professional athletes differs from that of more sedentary subjects in composition and particularly at the functional metabolic level. Gut. 2018;67:625–33. 28360096 10.1136/gutjnl-2016-313627

[52] O’Sullivan O, Cronin O, Clarke SF, Murphy EF, Molloy MG, Shanahan F, et al. Exercise and the microbiota Gut Microbes. 2015;6:131–6. 25800089 10.1080/19490976.2015.1011875 PMC4615660

[53] Wu L, Xie X, Li Y, Liang T, Zhong H, Yang L, et al. Gut microbiota as an antioxidant system in centenarians associated with high antioxidant activities of gut-resident Lactobacillus. Npj Biofilms Microbiomes. 2022;8:1–17. 36564415 10.1038/s41522-022-00366-0 PMC9789086

[54] Shandilya S, Kumar S, Kumar Jha N, Kumar Kesari K, Ruokolainen J. Interplay of gut microbiota and oxidative stress: perspective on neurodegeneration and neuroprotection. J Adv Res. 2022;38:223–44. 35572407 10.1016/j.jare.2021.09.005 PMC9091761

[55] Zheng Y, Zhang L, Bonfili L, de Vivo L, Eleuteri AM, Bellesi M. Probiotics supplementation attenuates inflammation and oxidative stress induced by chronic sleep restriction. Nutrients. 2023;15:1518. 36986248 10.3390/nu15061518 PMC10054086

[56] Hohor S, Mandanach C, Maftei A, Zugravu CA, Oțelea MR. Impaired melatonin secretion, oxidative stress and metabolic syndrome in night shift work. Antioxid Basel Switz. 2023;12:959. 10.3390/antiox12040959 PMC10135726 37107334

[57] Hill VM, O’Connor RM, Sissoko GB, Irobunda IS, Leong S, Canman JC, et al. A bidirectional relationship between sleep and oxidative stress in Drosophila. PLoS Biol. 2018;16: e2005206. 30001323 10.1371/journal.pbio.2005206 PMC6042693

[58] Radak Z, Taylor AW, Ohno H, Goto S. Adaptation to exercise-induced oxidative stress: from muscle to brain. Exerc Immunol Rev. 2001;7:90–107. 11579750

[59] Fukui T, Yamauchi K, Maruyama M, Yasuda T, Kohno M, Abe Y. Significance of measuring oxidative stress in lifestyle-related diseases from the viewpoint of correlation between d-ROMs and BAP in Japanese subjects. Hypertens Res. 2011;34:1041–5. 21677660 10.1038/hr.2011.76

[60] Kim JH, Baik HW, Yoon YS, Joung HJ, Park JS, Park SJ, et al. Measurement of antioxidant capacity using the biological antioxidant potential test and its role as a predictive marker of metabolic syndrome. Korean J Intern Med. 2014;29:31–9. 24574831 10.3904/kjim.2014.29.1.31 PMC3932393

[61] Vgontzas AN, Papanicolaou DA, Bixler EO, Lotsikas A, Zachman K, Kales A, et al. Circadian interleukin-6 secretion and quantity and depth of sleep. J Clin Endocrinol Metab. 1999;84:2603–7. 10443646 10.1210/jcem.84.8.5894

[62] Hong S, Mills PJ, Loredo JS, Adler KA, Dimsdale JE. The association between interleukin-6, sleep, and demographic characteristics. Brain Behav Immun. 2005;19:165–72. 15664789 10.1016/j.bbi.2004.07.008

[63] Rohleder N, Aringer M, Boentert M. Role of interleukin-6 in stress, sleep, and fatigue. Ann N Y Acad Sci. 2012;1261:88–96. 22823398 10.1111/j.1749-6632.2012.06634.x

[64] Saravanan P, R P, Balachander N, K KRS, S S, S R. Anti-inflammatory and wound healing properties of lactic acid bacteria and its peptides. Folia Microbiol (Praha). 2023;68(3):337–53. 10.1007/s12223-022-01030-y PMC9924211 36780113

[65] Yu L, Han X, Cen S, Duan H, Feng S, Xue Y, et al. Beneficial effect of GABA-rich fermented milk on insomnia involving regulation of gut microbiota. Microbiol Res. 2020;233: 126409. 31927503 10.1016/j.micres.2020.126409

[66] Ogawa Y, Miyoshi C, Obana N, Yajima K, Hotta-Hirashima N, Ikkyu A, et al. Gut microbiota depletion by chronic antibiotic treatment alters the sleep/wake architecture and sleep EEG power spectra in mice. Sci Rep. 2020;10:19554. 33177599 10.1038/s41598-020-76562-9 PMC7659342

[67] Abate M, Salini V. Oxidative Stress, Testosterone, Cortisol, and Vitamin D: Differences in Professional Soccer Players of African and Caucasian Origin. Med Princ Pract. 2022;31:352–8. 35764054 10.1159/000525728 PMC9485990

[68] Abate M, Carlo DI, L, Cocco G, Cocco A, Salini V. Testosterone, cortisol, vitamin D and oxidative stress and their relationships in professional soccer players. J Sports Med Phys Fitness. 2022;62:382–8. 34080814 10.23736/S0022-4707.21.12094-8

[69] Wu H-J, Wu E. The role of gut microbiota in immune homeostasis and autoimmunity. Gut Microbes. 2012;3:4–14. 22356853 10.4161/gmic.19320 PMC3337124

[70] Pedersen BK, Steensberg A, Schjerling P. Exercise and interleukin-6. Curr Opin Hematol. 2001;8(3):137–41. 11303145 10.1097/00062752-200105000-00002

