# Supplementary material for: A Lactobacillus consortium provides insights into the sleep-exercise-microbiome nexus in proof of concept studies of elite athletes and in the general population
Source: Microbiome. 2025 Jan 2;13:1. doi: 10.1186/s40168-024-01936-4 (PMC11697739; doi:10.1186/s40168-024-01936-4)

AEs reported during the open label study. Among those that completed the study, this bar graph shows AEs participants attributed to the probiotic (includes data for both week1 and week2)

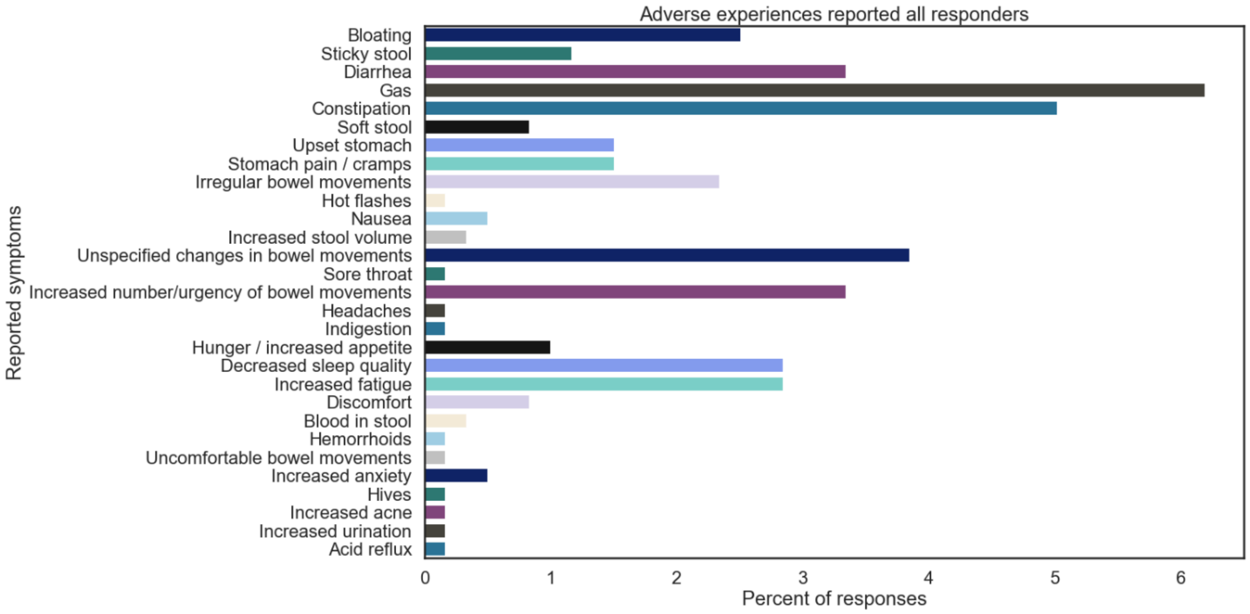

Supplement: Supplementary file 2 — Additional file 1: AEs reported during the open-label study. [file 40168_2024_1936_MOESM1_ESM.pdf]
