# Supplementary material for: A Lactobacillus consortium provides insights into the sleep-exercise-microbiome nexus in proof of concept studies of elite athletes and in the general population
Source: Microbiome. 2025 Jan 2;13:1. doi: 10.1186/s40168-024-01936-4 (PMC11697739; doi:10.1186/s40168-024-01936-4)
Supplement: Supplementary file 3 — Additional file 2: Top 10 most important features of the model. [file 40168_2024_1936_MOESM2_ESM.pdf]

Top 10 most important features of the model

**Feature**	**Importance**
Week 2 Change in general health and fitness	0.117058
Week 2 Change in bowel movements	0.059100
Week 2 Typical BM was normal	0.031619
Change in feeling during workout after week 2	0.029926
Age	0.028157
Change in fatigue frequency after week 2	0.025606
Week 2 Energy level	0.023331
Change in workout intensity after week 2	0.018657
Annual income ($)	0.018455
Week 2 Current health	0.015684
