# Supplementary material for: A Lactobacillus consortium provides insights into the sleep-exercise-microbiome nexus in proof of concept studies of elite athletes and in the general population
Source: Microbiome. 2025 Jan 2;13:1. doi: 10.1186/s40168-024-01936-4 (PMC11697739; doi:10.1186/s40168-024-01936-4)
Supplement: Supplementary file 4 — Additional file 3: Survey used in placebo-controlled study. [file 40168_2024_1936_MOESM3_ESM.pdf]

1. How would you rate your overall experience with Nella's athlete-derived performance probiotics?

   1 = Hated it
   2 = Did not enjoy it
   3 = Neutral
   4 = It was pretty good
   5 = Loved it

2. Looking back on this last week compared to your personal baseline before you started taking these probiotics, how do you feel your general health, digestion, and fitness has changed over the last month?

   1 = Significantly worse
   2 = Worse
   3 = No change
   4 = Better
   5 = Significantly better

3. How were the quality of your **bowel movements** this past week compared to your personal normal before taking these probiotics?

   1 = Significantly worse: Much more digestive issues or discomfort than usual
   2 = Worse
   3 = No change
   4 = Better
   5 = Significantly better: Much more regular, better formed stool, and / or fewer digestive issues than usual

4. How would you rate your **sleep quality** this past week compared to your personal normal before taking these probiotics?

   1 = Significantly worse: Did not get enough hours of sleep and / or have very disturbed restless sleep. Woke up feeling exhausted.
   2 = Worse
   3 = No change
   4 = Better
   5 = Significantly better: Got plenty of deep undisturbed sleep. Woke up feeling well rested.

5. How would you rate your **energy level** this past week relative to your personal normal before taking these probiotics?

   1 = Significantly worse: Less energy / more fatigued than usual
   2 = Worse
   3 = No change
   4 = Better
   5 = Significantly better: More energized than usual

6. How often did you experience **fatigue** this past week relative to your personal normal before taking these probiotics?

1 = Significantly worse: Much more fatigued than usual and can't manage to do the workouts I'm used to
2 = Worse
3 = No change
4 = Better
5 = Significantly better: Much less fatigued than usual with more energy every day

7. How did you feel **during** your workouts this past week compared to your personal normal before taking these probiotics?

   1 = Significantly worse: Weaker with more fatigue, lightheadedness, off balance, etc.
   2 = Worse
   3 = No change
   4 = Better
   5 = Significantly better: Stronger with more energy, focus, better form, etc.

8. How **sore** were you **after** your most intense workouts this week compared to your personal normal before taking these probiotics?

   1 = Significantly worse: Much more pain and soreness
   2 = Worse
   3 = No change
   4 = Better
   5 = Significantly better: Much less pain and soreness

9. How long did it take for you to **recover after** your most intense workouts this week compared to your personal normal before taking these probiotics?

   1 = Significantly worse: My muscles feel sore and fatigued for much longer than I'm used to.
   2 = Worse
   3 = No change
   4 = Better
   5 = Significantly better: My body is recovering quickly. I feel like I can do another intense workout much sooner than I used to.

10. At any point in the last month, did you purposely stop using the probiotics because of symptoms you experienced that you think were caused by the probiotic?

    Yes No

11. If you answered "Yes" to Question 10, can you describe in a few words, the symptoms that you had?

    ____

12. Do you believe taking these probiotics on a consistent basis will help improve your overall health and / or athletic performance?

Yes No
