# Supplementary material for: A Lactobacillus consortium provides insights into the sleep-exercise-microbiome nexus in proof of concept studies of elite athletes and in the general population
Source: Microbiome. 2025 Jan 2;13:1. doi: 10.1186/s40168-024-01936-4 (PMC11697739; doi:10.1186/s40168-024-01936-4)
Supplement: Supplementary file 5 — Additional file 4: Species with significant changes over time. [file 40168_2024_1936_MOESM4_ESM.pdf]

Longitudinal volatility analysis (Qiime2) identified 11 species whose abundance changes over time

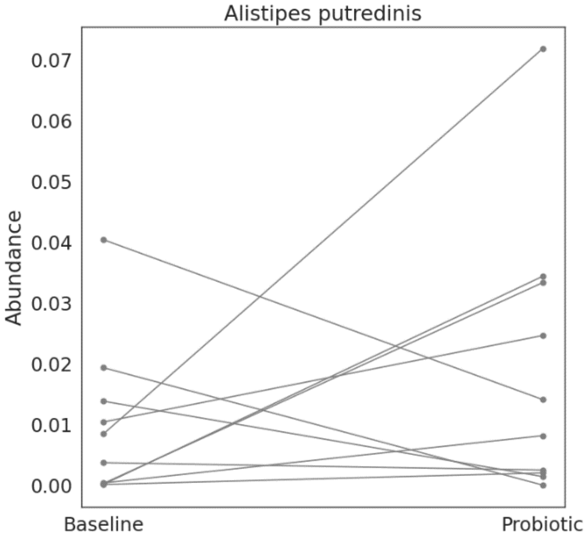

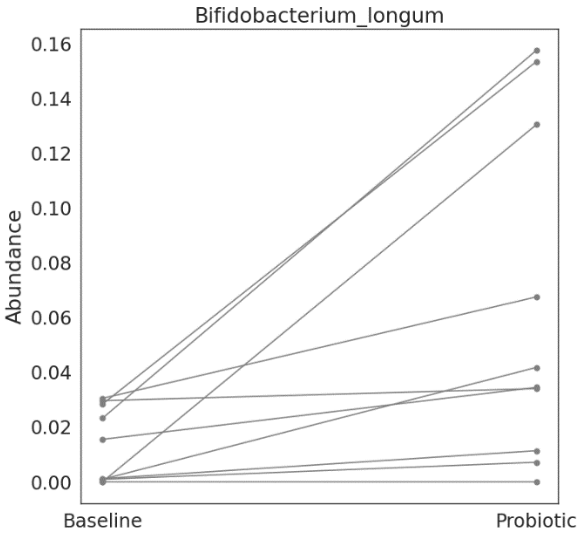

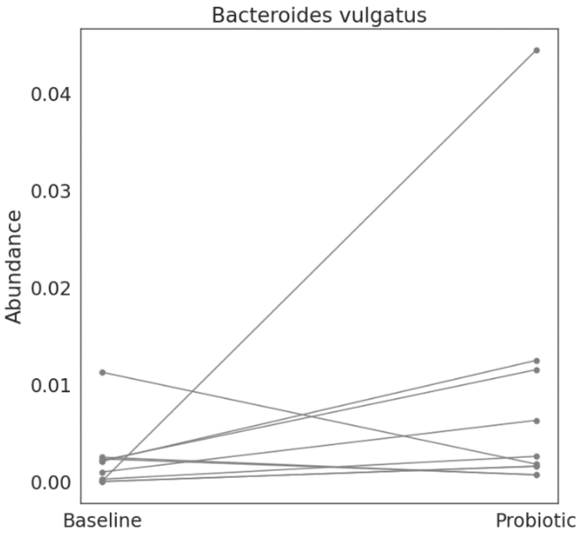

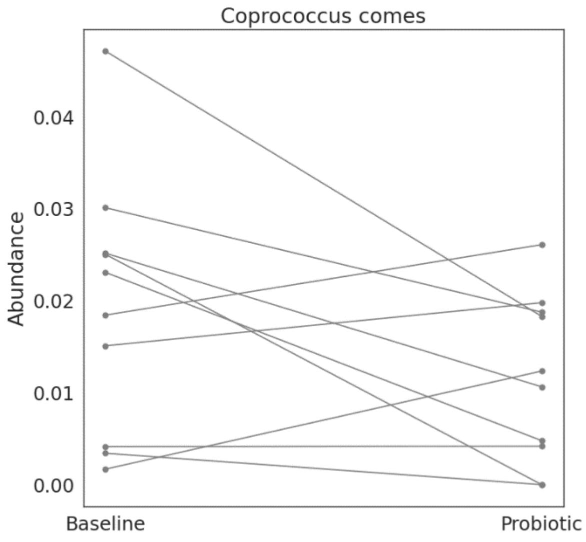

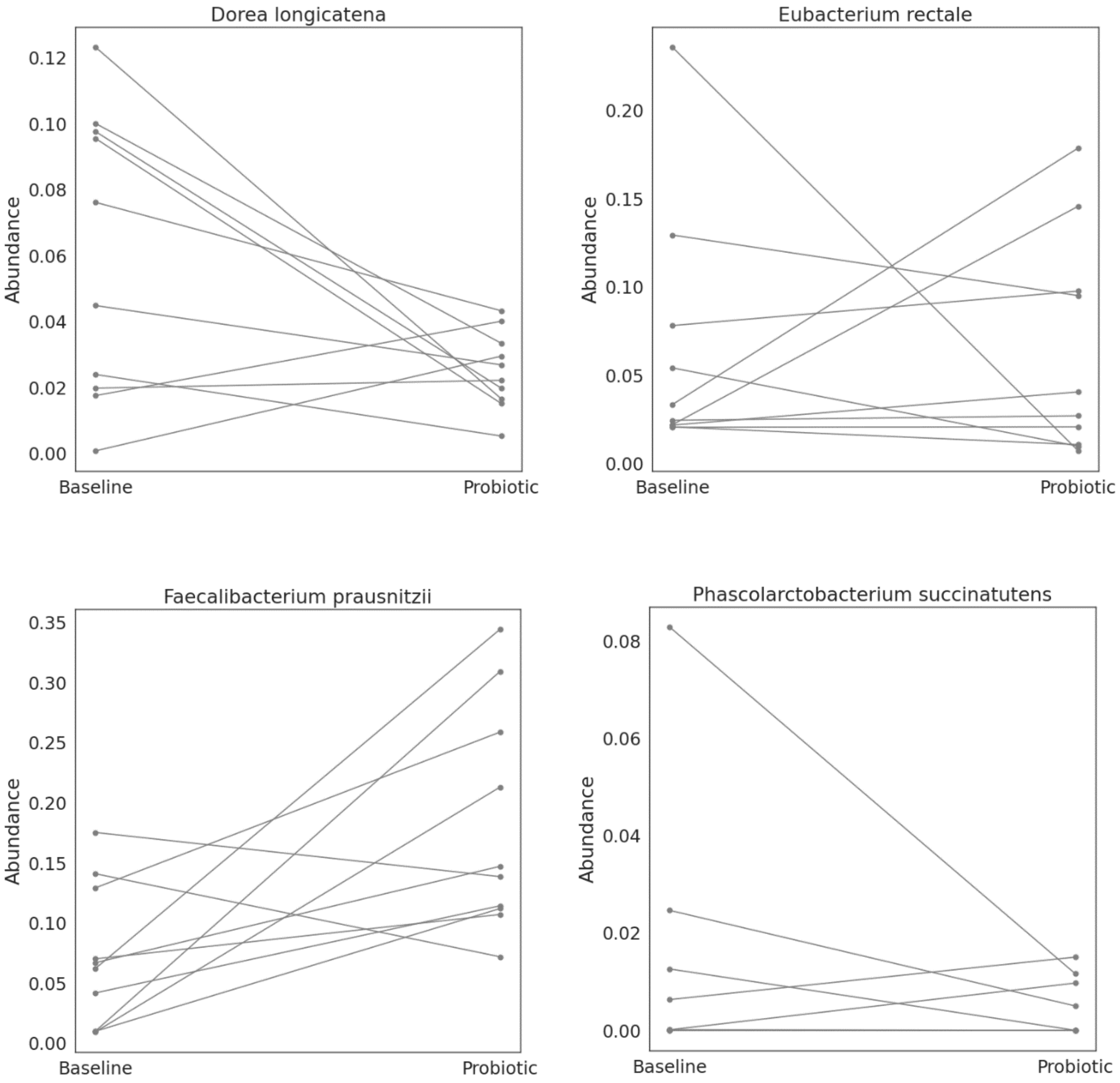
Dorea longicatena
Abundance
0.12
0.10
0.08
0.06
0.04
0.02
0.00
Baseline
Probiotic
Eubacterium rectale
Abundance
0.20
0.15
0.10
0.05
0.00
Baseline
Probiotic
Faecalibacterium prausnitzii
Abundance
0.35
0.30
0.25
0.20
0.15
0.10
0.05
0.00
Baseline
Probiotic
Phascolarctobacterium succinatutens
Abundance
0.08
0.06
0.04
0.02
0.00
Baseline
Probiotic

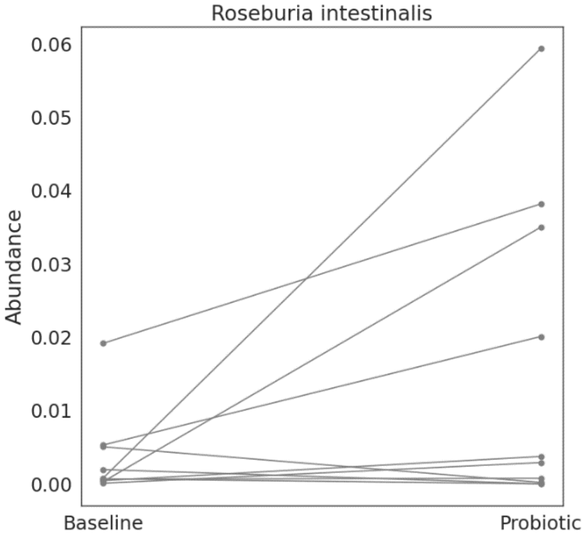
Roseburia intestinalis
Abundance
0.06
0.05
0.04
0.03
0.02
0.01
0.00
Baseline
Probiotic

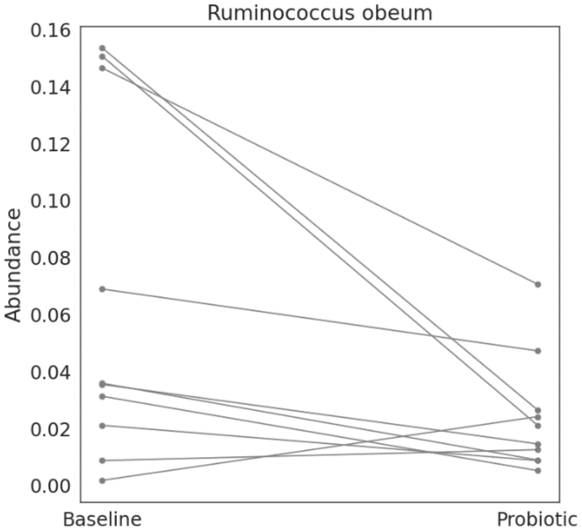
Ruminococcus obeum
Abundance
0.16
0.14
0.12
0.10
0.08
0.06
0.04
0.02
0.00
Baseline
Probiotic

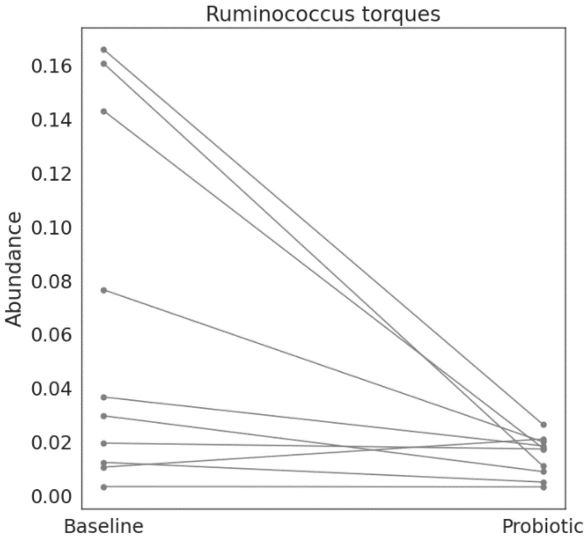
Ruminococcus torques
Abundance
0.16
0.14
0.12
0.10
0.08
0.06
0.04
0.02
0.00
Baseline
Probiotic
