# Supplementary figures and images for: A Lactobacillus consortium provides insights into the sleep-exercise-microbiome nexus in proof of concept studies of elite athletes and in the general population
Source: Microbiome. 2025 Jan 2;13:1. doi: 10.1186/s40168-024-01936-4 (PMC11697739; doi:10.1186/s40168-024-01936-4)

Clustering heatmap of all taxa, pathways, and metabolites

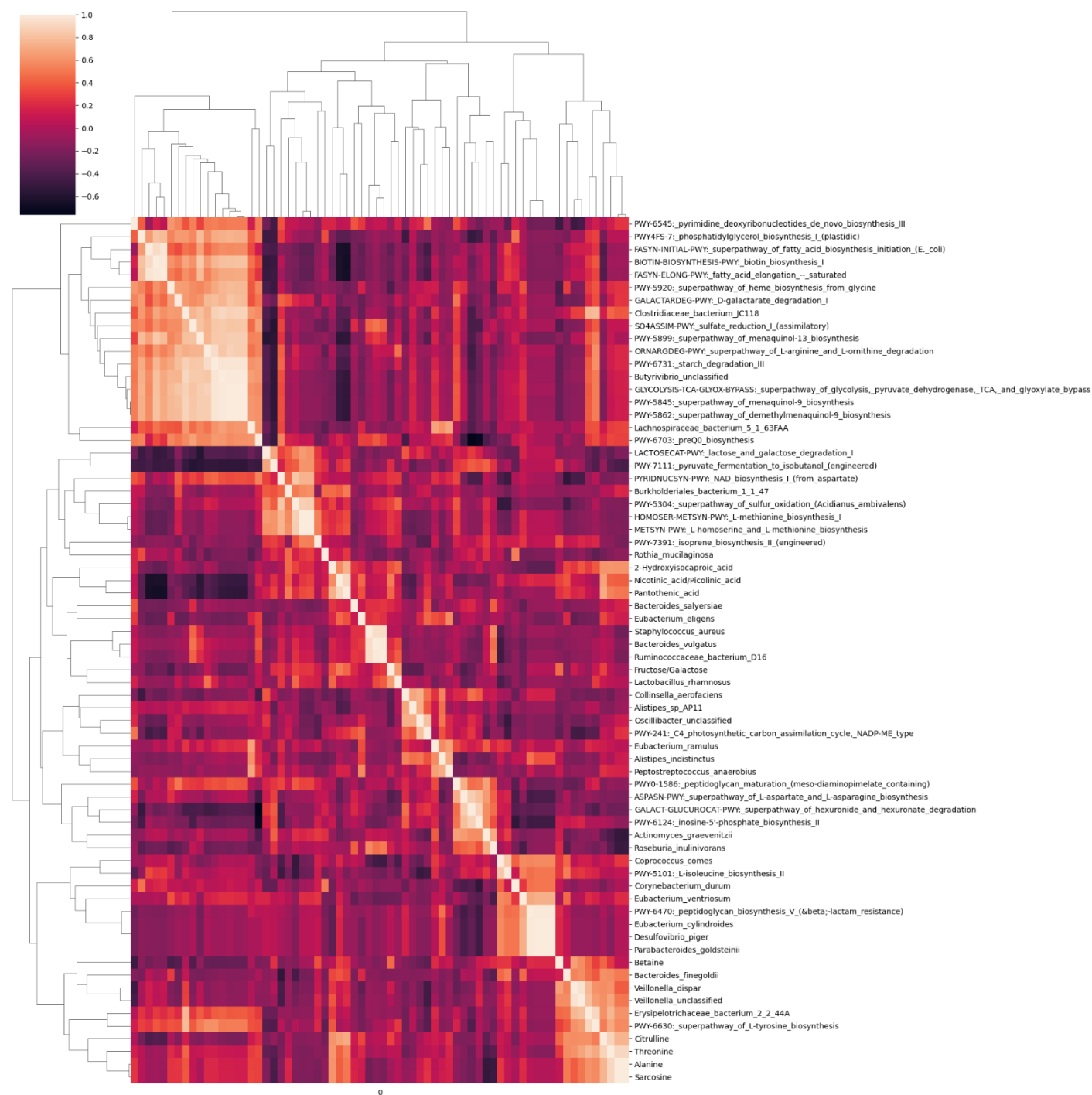

Supplement: Supplementary file 7 — Additional file 6: Clustering heatmap of all taxa, pathways, and metabolites. [file 40168_2024_1936_MOESM6_ESM.pdf]
